# Modelling mesenchymal stromal cell growth in a packed bed bioreactor with a gas permeable wall

**DOI:** 10.1371/journal.pone.0202079

**Published:** 2018-08-27

**Authors:** Michael J. Osiecki, Sean D. L. McElwain, William B. Lott

**Affiliations:** 1 Institute of Health and Biomedical Innovation, Queensland University of Technology, Brisbane, QLD, Australia; 2 School of Chemistry, Physics and Mechanical Engineering, Science and Engineering Faculty, Queensland University of Technology, Brisbane, QLD, Australia; 3 School of Mathematical Sciences, Queensland University of Technology, Brisbane, QLD, Australia; Stellenbosch University, SOUTH AFRICA

## Abstract

A mathematical model was developed for mesenchymal stromal cell (MSC) growth in a packed bed bioreactor that improves oxygen availability by allowing oxygen diffusion through a gas-permeable wall. The governing equations for oxygen, glucose and lactate, the inhibitory waste product, were developed assuming Michaelis-Menten kinetics, together with an equation for the medium flow based on Darcy’s Law. The conservation law for the cells includes the effects of inhibition as the cells reach confluence, nutrient and waste product concentrations, and the assumption that the cells can migrate on the scaffold. The equations were solved using the finite element package, COMSOL. Previous experimental results collected using a packed bed bioreactor with gas permeable walls to expand MSCs produced a lower cell yield than was obtained using a traditional cell culture flask. This mathematical model suggests that the main contributors to the observed low cell yield were a non-uniform initial cell seeding profile and a potential lag phase as cells recovered from the initial seeding procedure. Lactate build-up was predicted to have only a small effect at lower flow rates. Thus, the most important parameters to optimise cell expansion in the proliferation of MSCs in a bioreactor with gas permeable wall are the initial cell seeding protocol and the handling of the cells during the seeding process. The mathematical model was then used to identify and characterise potential enhancements to the bioreactor design, including incorporating a central gas permeable capillary to further enhance oxygen availability to the cells. Finally, to evaluate the issues and limitations that might be encountered scale-up of the bioreactor, the mathematical model was used to investigate modifications to the bioreactor design geometry and packing density.

## Introduction

For mesenchymal stem/stromal (MSC) cell-based therapy to become routine and economically viable, an automated closed-system bioreactor will be required to isolate and expand MSC populations, and many bioreactor designs have been described for this purpose [1–6]. Previous packed-bed bioreactor designs have required that essential nutrients and oxygen are efficiently supplied by medium perfusion alone. However, the shear stresses arising from mixing and medium perfusion in a packed bed bioreactor can compromise MSCs “stemness” during expansion and must be carefully modulated [7–10]. A shear stress of 0.015 Pa has been reported to up-regulate the osteogenic pathways in human bone marrow MSCs [7–9, 11]. Thus the scalability of packed-bed devices is limited by the maximum perfusion flow velocity, which cannot exceed 3 x 10^−4^ m/s without compromising the growth rate [9].

We recently developed a packed bed bioreactor design for the expansion of MSCs that decouples the medium nutrient supply from oxygen transport by using a gas-permeable wall to allow radial oxygen diffusion [12]. Oxygen is the limiting metabolite in bioreactors due to its low solubility in cell culture medium, and thus is the most difficult to adequately supply through perfusion. As the gas-permeable bioreactor no longer relies solely on oxygen supplied by the perfusion medium, the flow rate can be greatly reduced to control the glucose supply only. The gas-permeable bioreactor achieved similar MSC growth rates to other bioreactors reported in literature [1, 2, 13, 14], but the growth rate of the MSCs in the gas-permeable bioreactor was significantly less than observed in traditional tissue culture flasks. We hypothesised three factors that could contribute to this observation: (1) the supply of oxygen and glucose was inadequate, leading to significant concentration gradients within the scaffold, (2) the lower flow rate incorporated in the design insufficiently removed the lactate waste product [15, 16], and (3) a homogenous cell distribution was not achieved in the bioreactor. Initial heterogeneous cell distribution during seeding might affect the growth rate and thus the final cell number. Here we report a mathematical model developed to evaluate the role of these three factors and to direct future improvements to the bioreactor design.

Many perfusion models have been reported for cell expansion on a scaffold for tissue engineering purposes, and models designed to investigate cell growth that focus on nutrient concentration [17–19] and perfusion devices [20–23] formed the basis of our theoretical treatment. Based on previous experimental work [20], Lewis *et al*. modelled the interaction between oxygen profiles and cell distribution in a cartilaginous tissue construct [19]. Their cell growth and uptake description were based on early tumour models [17, 24] using Michaelis-Menten kinetics for cellular uptake of oxygen. However, their model did not consider cellular migration on the solid substrate or any advective transport of nutrients, oxygen and waste products. Their simulation predicted a high cell proliferation around the outside of the scaffold with a region of hypoxia in the centre. Zhao *et al*. [23] showed the importance of perfusion on MSC growth on a polyethylene-terephthalate scaffold in a perfusion bioreactor, achieving four times the cell density compared to static culture conditions. The 2-dimensional oxygen concentration profile model suggested the importance of perfusion, with far less depletion of oxygen within the scaffold and a decreased oxygen gradient compared to the static culture. However, the cell growth rate in their model was constant, and the effects of oxygen concentration or cell migration on the cell density were not considered. Coletti *et al*. [21] developed a more comprehensive 2-dimensional axisymmetric model that included advection and the diffusive transport of oxygen. The culture medium flow within the bioreactor was modelled in two ways, as there was a channel around the scaffold in the centre of the bioreactor. The medium flow outside the scaffold was described by the Navier-Stokes equation for an incompressible fluid, while the flow through the scaffold was described by the Brinkman’s extension to Darcy’s Law. The cellular nutrient uptake was modelled using Michaelis-Menten kinetics. The cellular growth rate was assumed to be described by the Contois equation, which considers the nutrient concentration and the cell density. However, cell migration was ignored. Chung *et al*. [25] further developed this bioreactor model that used Michaelis-Menten kinetics for cellular uptake of nutrients, as well as a modified Contois equation for the cellular growth, to include cell migratory terms. They also explored the effect of scaffold permeability and porosity on cell growth. In their model, the cell growth caused a reduction in scaffold pore size that changed the flow through the scaffold and increased shear stress. This reduction in nutrient supply to the cells impacted the growth rate. Since the Reynolds number is small, Shakeel *et al*. [26] reduced the complexity of the fluid dynamic system by neglecting the inertial forces of the fluid flow and used Darcy’s Law to describe the fluid flow through the scaffold [27]. Their model suggested that both shear stresses and reduction in pore size compromised cell growth [22, 26]. They also investigated the importance of the initial seeding density distribution on the final total cell number by assuming that all the cells were attached either in a central region or along the walls of the scaffold.

The above models accounted for the effect of only one limiting metabolite (either oxygen or glucose) on the cell growth rate. However, the low flow rate in our gas-permeable bioreactor design required considering the effects of both oxygen and glucose depletion on the cell growth rate. In addition, the lower flow rate reduced the removal rate of the lactate waste product, which is known to reduce the growth rate of cells [15, 16]. The Monod model of mammalian cell growth describes the limiting effects of insufficient oxygen and glucose concentrations plus the inhibitory effect of lactate accumulation [28–30]. Unfortunately, accounting for the complexities of cell metabolism in a mathematical model can be difficult. For example, the rate of glucose consumption and the rate of lactate production can be hypothetically related by the stoichiometric ratio of lactate-to-glucose [15]. Although both glucose consumption and lactate production are much higher under perfusion conditions than under static conditions due to the increased cell growth rate, the lactate-to-glucose ratio is lower under perfusion conditions. This observation in MSCs suggests increased aerobic respiration relative to their preferred anaerobic respiration, which is possibly due to an increased oxygen supply during perfusion that prevents regions of hypoxia [23].

In most bioreactor designs, medium perfusion is the sole source of oxygen. The flow rate must increase as the cell number increases in these devices to prevent oxygen depletion and to maintain a near-homogenous concentration of other nutrients. As oxygen is the limiting metabolite due to poor solubility, decoupling the oxygen supply from the bulk medium flow will allow for lower perfusion rates without compromising cell growth. To achieve this, oxygen can by supplied by diffusion through interwoven gas permeable fibres in a hollow fibre bioreactor [31] or through a micro-bioreactor wall made of a gas permeable polymer [32]. Kim, *et al*. [33] modelled the oxygen pressure drop in a microfluidic device where the only source of oxygen was through a diffusive PDMS wall. The 2-dimensional diffusive model explored the role of the thickness of the PDMS on cellular density and the time required to reach oxygen equilibrium in the medium. Depending on the cellular uptake rate, they predicted that the cell density will be affected by a wall thickness greater than 5 mm with an effective diffusion time of 26 minutes, suggesting that diffusion resistance becomes high for a microfluidic scale device.

We have developed 2-dimensional axisymmetric model for MSC growth in our oxygen-decoupled packed bed bioreactor. The model included equations for oxygen, glucose and lactate transport in the scaffold regarded as flow through a porous medium. The model includes the effects of oxygen transport through the gas permeable wall of the bioreactor and was used to predict the dependence of the cellular growth rate on the inlet nutrient concentration and on the initial cell distribution. This model was initially developed to help interpret the experimental observation that the cell yield in the oxygen-decoupled bioreactor was less than the cell yield in a traditional tissue culture flask [12]. The effects of scale-up of our bioreactor were then evaluated by increasing the assumed vessel dimensions and by decreasing the particle size of the packed-bed, thus increasing the scaffold surface area. The finite element solver package COMSOL numerically solved the model equations.

### Model description

**Fig 1:**
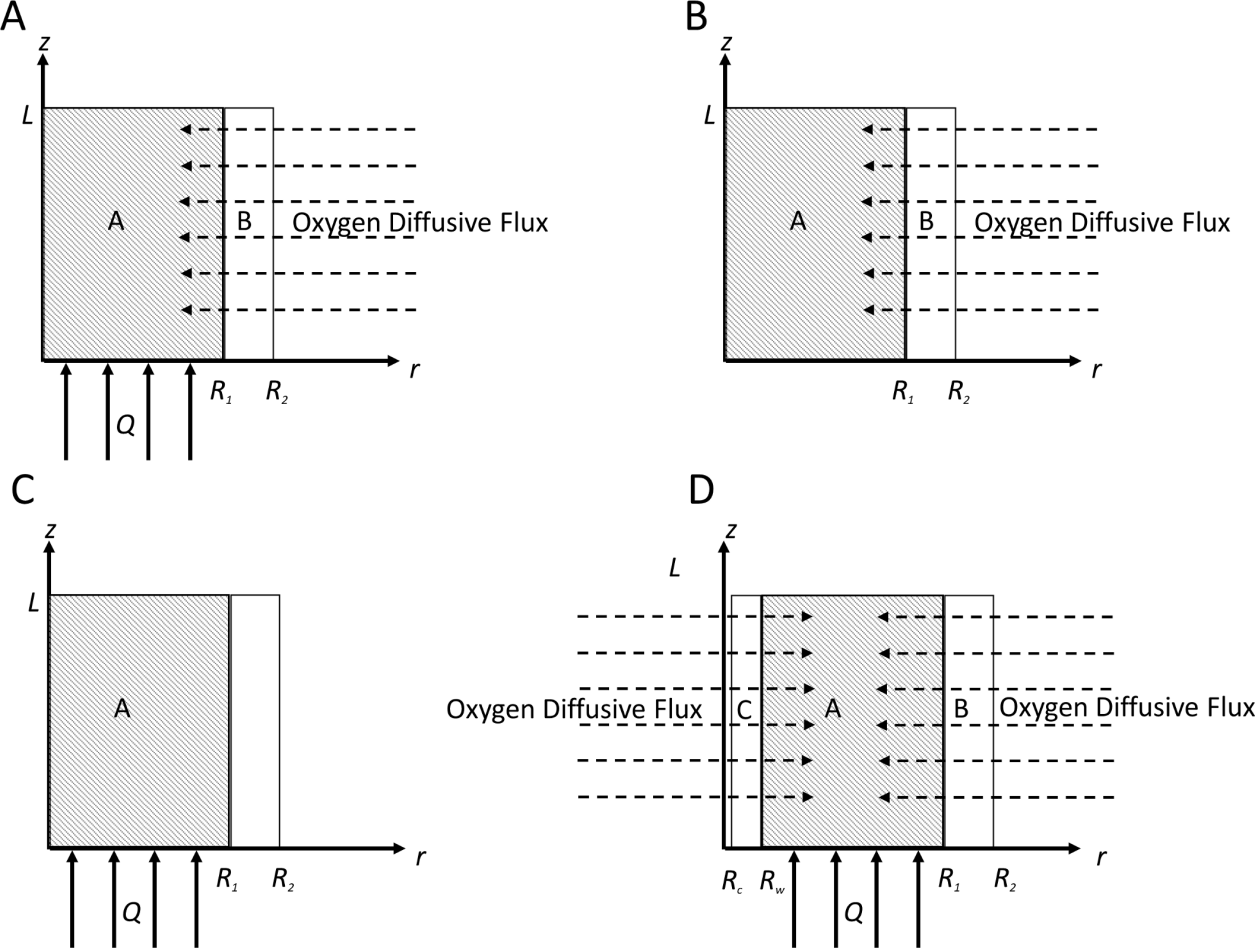
(A) Axis of symmetry diagram of perfusion bioreactor with a gas permeable wall. Oxygen is allowed to diffuse radially into the bioreactor. *R*_*2*_ is the radius of the PDMS cylinder, *R*_*1*_ is the radius of the scaffold volume of the bioreactor, and *L* is the length. In the equation development, the radial coordinate was denoted by *r* and the longitudinal coordinate by *z* (measured from the inlet). (B) The experiments were also performed in a batch configuration where the medium was replaced every two days, however oxygen could still diffuse through the wall. (C) Traditional packed/fixed bed bioreactor where all the nutrients and oxygen are provided by medium perfusion alone. (D) Further enhancement of the design by incorporating a central capillary with outer wall radius *R*_*w*_ and lumen *R*_*c*_ in the centre of the reactor provides an additional oxygen source.

For the purpose of mathematical modelling, the cells were assumed to be initially seeded with a uniform density in the porous scaffold. The scaffold with a radius R_1_ and a length L consisted of a randomly-packed bed of polystyrene cylinders with 2.5 mm diameter by 3 mm in length. The pore size in the scaffold was assumed to be unchanged by the presence of a confluent cell layer or any extracellular matrix components excreted during expansion. This assumption is reasonable as the maximum cell thickness of 1–2 μm is small compared to the scaffold pore size of greater than 1 mm. Under these conditions, the porosity and permeability of the scaffold is considered constant. The fluid (cell culture medium) was assumed to be viscous, incompressible and Newtonian. It was pumped into the scaffold at the boundary z = 0 and leaves at boundary z = L (Fig 1). The fluid (medium) was pumped at a constant volumetric flow rate *Q*. The medium contains oxygen and glucose, which are consumed by the cells. A small amount of lactate is present initially in the cell culture medium associated with the addition of foetal bovine serum. The gas permeable polymer bioreactor wall was constructed from polydimethylsiloxane (PDMS) of a thickness R_2_-R_1_, which allowed the radial diffusion of oxygen through the bioreactor wall. The oxygen solubility in the PDMS was greater than that of the medium.

The subsequently described base model equations were modified to explore a batch configuration (Fig 1B), which was used to compare our oxygen-decoupled bioreactor design to a traditional packed-bed reactor (Fig 1C). The potential for simple oxygen diffusion through the bioreactor wall to be insufficient for a larger scale oxygen-decoupled bioreactor design required that additional sources of oxygen to be considered in the scaled-up bioreactor design. Additional oxygen delivered through a central gas-permeable capillary was modelled to evaluate the bioreactor scalability (Fig 1D).

### Development of model equations

The model leads to six partial differential equations. The first characterises the fluid flow through the porous material with velocity (*U*) driven by the pressure (*P*) and The partial differential equations describing the mass balance for the convective and diffusive transport of the nutrients within the bioreactor; oxygen (*C*_*O*,*A*_) and glucose (*C*_*G*_), and the waste product lactate (*C*_*L*_). The fifth equation describes oxygen diffusion through the wall (*C*_*O*,*B*_). The final equation is a conservation equation for the cell density (N) that includes terms describing the proliferation and migration within the scaffold.

### Fluid flow through the scaffold

The fluid velocity through the bioreactor scaffold is small to allow inertial forces to be neglected, thus the fluid flow can be regarded as flow through a porous medium given by Darcy’s Law. This relates the fluid velocity to the interstitial pressure as a function of the material permeability (K*) and the fluid dynamic viscosity *μ*. Darcy’s Law is defined as

$$U=-\frac{K^{*}}{\mu }\nabla P,$$ (1)

Where

$$K^{*}=\frac{d_{p}^{2}\Phi^{3}}{180{\left(1-\Phi \right)}^{2}}.$$ (2)

Assuming that the cells occupy an insignificant fraction of the scaffold pore space, the permeability (K*) is taken to be constant and is defined in terms of the particle diameter equivalent to a sphere (*d*_*p*_) and the scaffold porosity (Φ) [27]. The continuity equation the fluid, which is assumed to be incompressible, is

∇.U=0. (3)

The following boundary conditions are applied. As the fluid is viscous, there is no slip along the bioreactor wall, and axisymmetric flow is assumed, so that

$$\left\{ \begin{array}{c} U_{r}=0\\ U_{z}=0 \end{array}\;\mathrm{at}\;r=0\;\mathrm{and}\;R_{1},\;0<z<L.\right.$$ (4)

The flow at the entrance leads to

$$U_{z}=\frac{Q}{\pi R_{1}^{2}}\;\mathrm{at}\;0<r<R_{1},\;z=0,$$ (5)

where Q is the constant fluid flow rate (cm^3^/hr)

### Development of the nutrient conservation equation

Cells require oxygen and glucose to survive and proliferate. Lactate is produced by the metabolism of glucose and other metabolites and inhibits cell survival and proliferation. The transport equation for the nutrients includes terms for advection, diffusion and consumption of glucose and oxygen and a production term for lactate. Both glucose (C_G_) and lactate (C_L_) have similar conservation of mass equations and boundary conditions. However, as lactate is a product of glucose metabolism, we relate the lactate production rate to glucose consumption rate by simply multiplying by the lactate yield from glucose (Y_L/C_) [15, 16, 28]. We obtain the following transport equations:

$$\frac{{\partial C}_{G}}{\partial t}=D_{G}\nabla^{2}C_{G}-\nabla .(UC_{G})-\frac{V_{max,G}NC_{G}}{K_{m,G}+C_{G}},$$ (6)

and

$$\frac{{\partial C}_{L}}{\partial t}=D_{L}\nabla^{2}C_{L}-\nabla .(UC_{L})+Y_{L/C}\frac{V_{max,G}NC_{G}}{K_{m,G}+C_{G}}$$ (7)

The consumption of glucose is modelled assuming Michaelis-Menten kinetics. This is a function of the maximum uptake/production rate (V_max,I_, mM/hr/cell), the Michaelis-Menten constant (K_m,I_, mM), the cell density (N, cells/cm^3^) and the concentration (C_i_). The diffusion coefficient must be modified to account for the restriction to diffusion associated with a bioreactor that contains non-gas-permeable pellets that retard the overall diffusion. This modification is defined by the voidage (*ε*), tortuosity (*τ*) and the free diffusion coefficient (*D*_*m*,*i*_). The tortuosity is defined in (9) as a function of the porosity and the sphericity (*φ*) of the particles [34]. The sphericity of non-spherical particle is given by (10) as a function of the number of the particles per unit volume of the packed bed (n), the porosity, and the surface area to volume ratio (a) of the packed bed. Defined by

$$D_{i}=\frac{\varepsilon }{\tau }D_{m,i},$$ (8)

where

$$\tau =\frac{1.23{\left(1-\varepsilon \right)}^{\frac{4}{3}}}{\varepsilon \times \varphi^{2}},$$ (9)

and

$$\varphi =\frac{(36\times \pi \times n{\left(1-\varepsilon \right)}^{2}{)}^{\frac{1}{3}}}{a}$$ (10)

The concentrations of glucose and lactate are assumed to be constant at the inlet, and the longitudinal diffusive flux is assumed to be zero at the outlet and through the reactor wall. The symmetry condition gives a zero radial flux for all species at r = 0. These boundary conditions lead to

$$C_{i}=C_{in,i}\;\mathrm{at}\;z=0,\;0<r<R_{1},$$ (11)

$$\frac{\partial C_{i}}{\partial z}=0\;\mathrm{at}\;z=L,\;0<r<R_{1},$$ (12)

$$\frac{\partial C_{i}}{\partial r}=0\;\mathrm{at}\;r=R_{1},\;0<z<L,$$ (13)

$$\frac{\partial C_{i}}{\partial r}=0\;\mathrm{at}\;r=0,\;0<z<L.$$ (14)

Oxygen conservation leads to two equations, one to define the oxygen concentration in the scaffold (15) and the other for the oxygen diffusion in the wall (16). These equations are given by

$$\frac{{\partial C}_{O,A}}{\partial t}=D_{O}\nabla^{2}C_{O,A}-\nabla .(UC_{O,A})-\frac{V_{max,O}NC_{O,A}}{K_{m,O}+C_{O},A}\;\mathrm{for}\;0<r<R_{1}$$ (15)

$$\frac{\partial C_{O,B}}{\partial t}=D_{PDMS}\nabla^{2}C_{O,B}\;\mathrm{for}\;R_{1}<r<R_{2}$$ (16)

For Eq (15), the boundary conditions are set to exclude flux through inlet and outlet walls with constant concentration of oxygen at the outer wall. This is defined as the solubility of oxygen in PDMS (S_PDMS_, mM)

$$\frac{\partial C_{O,B}}{\partial z}=0\;\mathrm{at}\;z=0,\;L,\;R_{1}<r<R_{2},$$ (17)

$$C_{O,B}=S_{PDMS}\;\mathrm{at}\;r=R_{2},\;0<z<L.$$ (18)

At the interface between the wall and the scaffold, we require continuity of the diffusive flux and a change in concentration due to the difference in solubilities. The solubility of oxygen in the fluid is denoted by S_M_ (mM). The boundary conditions are

$$D_{O}\frac{\partial C_{O,A}}{\partial r}=D_{PDMS}\frac{\partial C_{O,B}}{\partial r}\;\mathrm{at}\;r=R_{1},\;0<z<L$$ (19)

and

$$\frac{C_{O,A}}{S_{M}}=\frac{C_{O,B}}{S_{PDMS}}\;\mathrm{at}\;r=R_{1},\;0<z<L$$ (20)

Within the scaffold itself, the fluid pumped into inlet is oxygenated, there is no diffusive flux at the outlet and at the axis of symmetry implies

$$C_{O,A}=S_{M}\;\mathrm{at}\;z=0,\;0<r<R_{1},$$ (21)

$$\frac{\partial C_{O,A}}{\partial z}=0\;\mathrm{at}\;z=L,\;0<r<R_{1},$$ (22)

$$\frac{\partial C_{O,A}}{\partial r}=0\;\mathrm{at}\;r=0,\;0<z<L.$$ (23)

### Development of cell growth equation

The local cell density changes over time, due to proliferation and the cells are free to migrate on the pellets of the scaffold. The proliferation rate is taken to be a logistic kinetic form with maximum cell density (N_max_, cell/cm^3^) and an intrinsic growth rate (k, hr^-1^) [17]. The growth rate dependence is taken as a Monod kinetic equation that is dependent on the nutrient concentrations (C_i_), maximum growth (K_max_, hr^-1^) and the Michaelis-Menten constants (K_G_, K_O_ and K_L_, mM) [18, 28]. The migration is governed by the diffusion coefficient (D_N_) and the cell density, so that

$$\frac{\partial N}{\partial t}=kN\;\left(1-\frac{N}{N_{max}}\right)+D_{N}\nabla^{2}N,$$ (24)

where

$$k=K_{max}(\frac{C_{G}}{K_{G}+C_{G}}\frac{C_{O,A}}{K_{O}+C_{O,A}}\frac{K_{L}}{K_{L}+C_{L}}).$$ (25)

The boundary conditions for the cell growth is derived as follows. The cells cannot leave the scaffold and r = 0 is an axis of symmetry so that

$$\frac{\partial N}{\partial z}=0\;\mathrm{at}\;z=L\;\mathrm{and}\;z=0,\;0<r<R_{1},$$ (26)

and

$$\frac{\partial N}{\partial r}=0\;\mathrm{at}\;r=R_{1},\;0<z<L,$$ (27)

with

$$\frac{\partial N}{\partial r}=0\;\mathrm{at}\;r=0,\;0<z<L.$$ (28)

#### Shear stress

The shear stress (*σ*, Pa) acting on the cells can be estimated from the Darcy velocity (U), porosity (ε), tortuosity (τ), viscosity (μ) of the medium and the pore diameter of the packed bed (D_H_) as follows

$$\sigma =\frac{8\mu \tau \|U\|}{D_{H}K^{*}},$$ (29)

The pore diameter is a function of the voidage and the particle diameter [35] so that

$$D_{H}=\frac{2\epsilon d_{p}}{3(1-\varepsilon )}.$$ (30)

### Initial conditions and parameters

Initially the cells are seeded with a uniform density of 1000 cells/cm^2^, which must be converted to cells per unit volume by multiplying the surface area to volume ratio (*a*), so that

$$N\left(r,z,0\right)=N_{i}\;\mathrm{at}\;0<r<R_{1}\;\mathrm{and}\;0<z<L.$$ (31)

The oxygen concentration in the medium and the PDMS are initially assumed to be in equilibrium with the surrounding atmosphere so that

$$C_{O,A}\left(r,z,0\right)=S_{M}\;\mathrm{at}\;0<r<R_{1}\;\mathrm{and}\;0<z<L,$$ (32)

and

$$C_{O,B}\left(r,z,0\right)=S_{PDMS}\;\mathrm{at}\;R_{1}<r<R_{2}\;\mathrm{and}\;0<z<L.$$ (33)

The reactor is initially filled with cell culture medium so that

$$C_{G}\left(r,z,0\right)=C_{in,G}\;\mathrm{at}\;0<r<R_{1}\;\mathrm{and}\;0<z<L,$$ (34)

and

$$C_{L}\left(r,z,0\right)=C_{in,L}\;\mathrm{at}\;0<r<R_{1}\;\mathrm{and}\;0<z<L.$$ (35)

Table 1 summarises the input parameters for the model, with * representing a measured value from our experiments [12].

**Table 1:** Model parameters and values used in model, * denotes measured values.

**Parameter**	Description	Value	Units	Ref
**Bioreactor Parameters**				
*R*_*1*_	Inner wall radius	0.75	cm	*
*R*_*2*_	Outer wall radius	1.25	cm	*
*L*	Scaffold length	7.5	cm	*
	Growth area of scaffold	158.8	cm^2^	*
	Scaffold volume	13.25	cm^3^	
***ε***	Voidage	0.47	-	*
**Φ**	Porosity	0.47	-	*
*A*	Surface area to volume ratio of the scaffold	12.013	cm^-1^	*
*N*	Number of particles per volume of packed bed	36	cm^-3^	*
*d*_*p*_	Particle diameter (equivalent to a sphere)	0.304	cm	*
*Q*	Medium flow rate	0.208	cm^3^/h	*
**Wall Properties**				
*S*_*PDMS*_	Solubility of oxygen in PDMS	1.3	mM	[36]
*D*_*PDMS*_	Oxygen diffusion coefficient in PDMS	5x10^-5^	cm^2^/s	[23]
**Fluid Properties**				
**S_m_**	Solubility of oxygen in medium	0.2	mM	*
**C_in,G_**	Initial glucose concentration	4.81	mM	*
**C_in,L_**	Initial lactate concentration	1.63	mM	*
**D_m,O_**	Oxygen diffusion coefficient in medium	3.290x10^-5^	cm^2^/s	[23]
**D_m,G_**	Glucose diffusion coefficient in medium	5.4x10^-6^	cm^2^/s	[37]
**D_m,L_**	Lactate diffusion coefficient in medium	1.1x10^-5^	cm^2^/s	[38]
***ρ***	Density of water @ 37°C	0.9933	g/cm^3^	[35]
***μ***	Viscosity of water @ 37°C	0.00692	kg/(m•s)	[35]
**Cell Properties**				
*N*_*max*_	Maximum cell density	23,000	cells/cm^2^	*
*N*_*i*_	Initial cell value	12013	cells/cm^3^	*
*D*_*N*_	Diffusion coefficient for cell movement	1.38x10^-10^	cm^2^/s	[39]
*V*_*max*,*O*_	Maximum uptake rate of oxygen	100	fmol/h/cell	[40]
*V*_*max*,*G*_	Maximum uptake rate of glucose	272	fmol/h/cell	[40]
*Y*_*L/G*_	Yield of lactate from glucose	2.0	-	[15]
*K*_*m*,*O*_	Michaelis-Menten constant for oxygen uptake	4.05x10^-9^	mol/cm^3^	[41]
*K*_*m*,*G*_	Michaelis-Menten constant for glucose uptake	3.5x10^-7^	mol/cm^3^	[42]
*K*_*max*_	Max growth rate	0.02631	hr^-1^	*
*K*_*O*_	Oxygen Michaelis-Menten growth constant	0.001	mM	[43]
*K*_*G*_	Glucose Michaelis-Menten growth constant	0.006	mM	[43]
*K*_*L*_	Lactate Michaelis-Menten growth constant	43	mM	[15]

### Solution method

The modelling leads to six partial differential equations representing fluid flow through the scaffold (1), nutrient transport of glucose (6), lactate (7), oxygen (15 & 16) and the cell density (24). Due to the complexities of the system, an analytical solution cannot be found. The commercially available finite element software COMSOL (vers. 4.4) solved the governing equations.

A free triangular mesh was generated and refined iteratively until a convergent result was achieved (S1 File). The refined mesh consisted of 9,628 elements with 500 boundary elements. The dependent variables were approximated by a quadratic shape function and the system was solved for 43,493 degrees of freedom. The time step was set to 0.1 hr for a total of 8 days (192 hr).

### Statistics

To quantify the fit of the model to experiment results the root-mean-square error (RMSE) was calculated.

## Results and discussion

### Base case-decoupling the oxygen supply from the bulk medium is superior to a traditional packed bed bioreactor

In experimental studies of the oxygen-decoupled bioreactor, a significant reduction in the MSC growth in the bioreactor compared to cells grown in a traditional T175 flask was observed [12]. The axisymmetric model of our oxygen-decoupled bioreactor was developed to explore this phenomenon and to further develop and optimise this bioreactor design.

**Fig 2:**
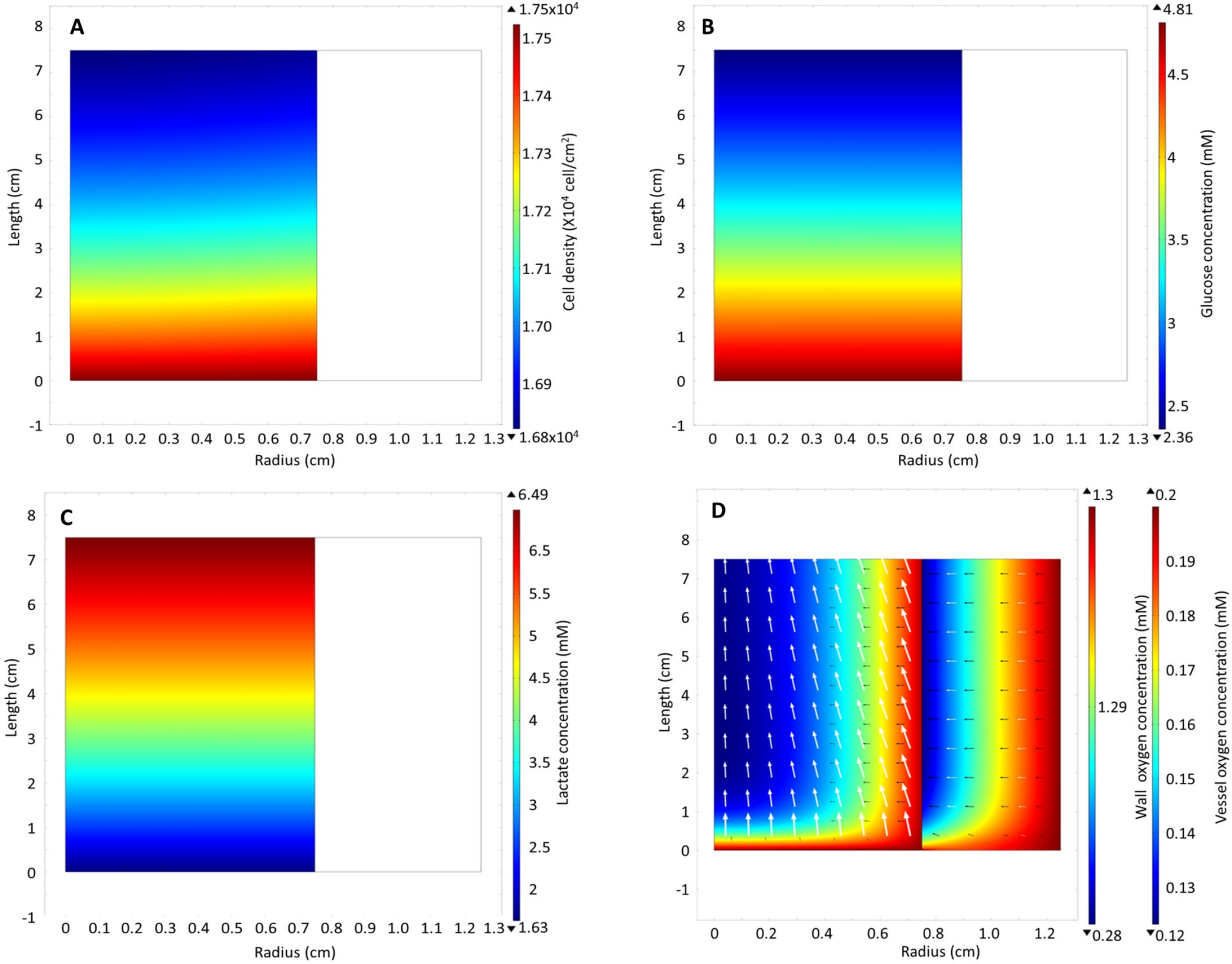
Model results: heat map of (A) cell density, (B) glucose, (C) lactate and (D) oxygen concentration in the wall and in the vessel on day 7. The white arrows on the oxygen concentration heat map represent the direction of total flux of oxygen while the black arrows represent the direction of the diffusive flux.

In our model, medium was perfused at a flow rate of 0.208 ml/hr, which putatively provides sufficient glucose to prevent depletion. Sufficient oxygen was assumed to be supplied by radial diffusion through the bioreactor wall. An initial uniform cell distribution was predicted to result in a final non-uniform cell distribution by the end of the culture period (Fig 2A). The large difference in cell density predicted between the inlet and outlet with only a small difference in the cell density in the radial direction suggested that the cell density gradient was driven strongly by glucose and/or lactate concentrations, rather than by oxygen availability. Driven by cell growth, these concentration differences and cell gradients increased over time as a result of nutrient consumption and lactate production (see S2–S5 File). The 51% reduction in the glucose concentration at the outlet compared to the inlet (Fig 2B) correlated with a similar increase in the lactate concentration (Fig 2C). Nevertheless, the model supports the hypothesis that radial diffusion of oxygen is sufficient to support cell growth in the oxygen-decoupled bioreactor without depleting oxygen in the centre of the bioreactor (Fig 2D).

**Fig 3:**
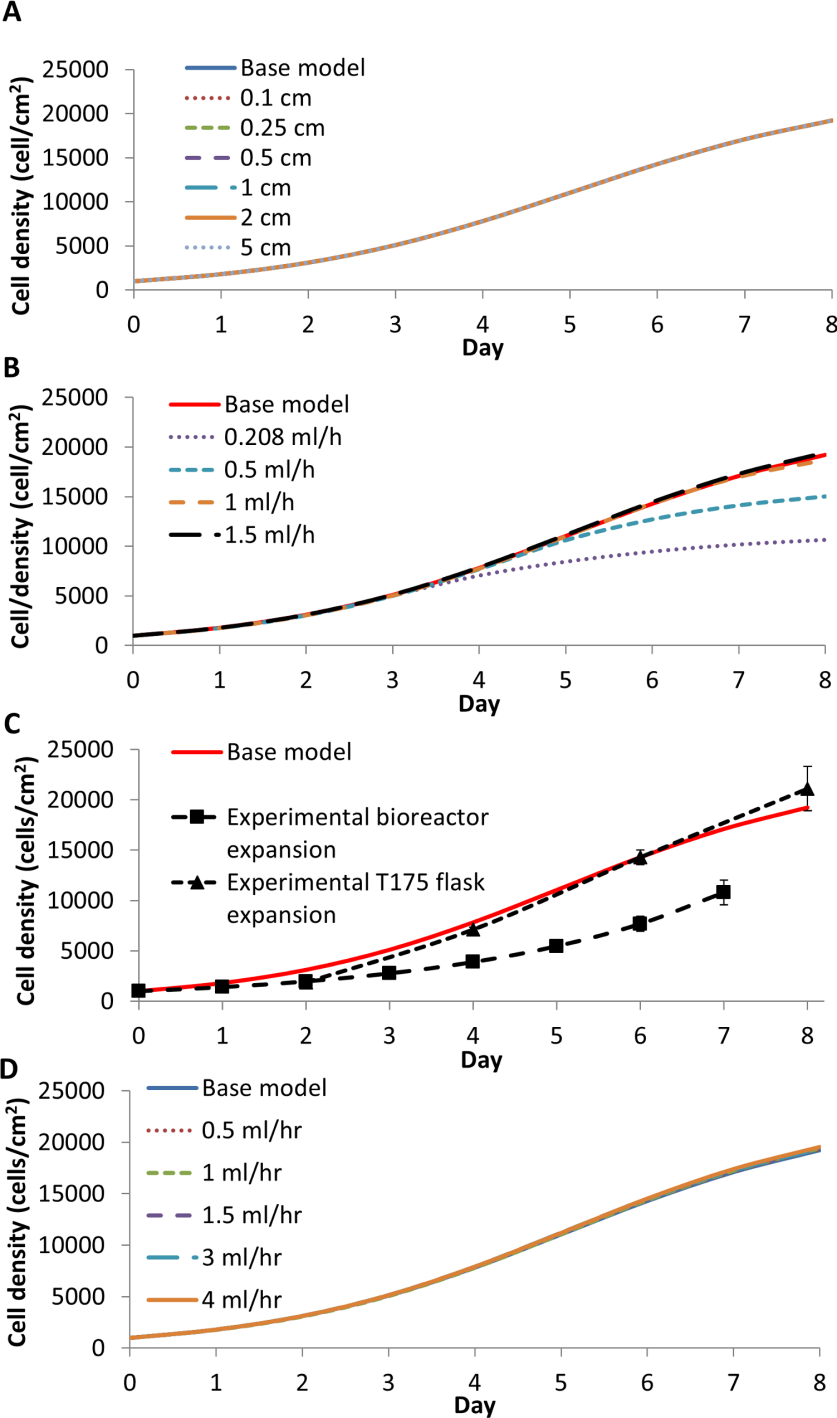
(A) The predicted average cell density change as a result of increasing the wall thickness from 0.1 to 5 cm. (B) A bioreactor model average cell density without wall diffusion requires a higher medium flow rate compared to our bioreactor with wall diffusion (Base model). (C) The average cell densities achieved experimentally from T175 flask and bioreactor expanded cells were compared to base model output. Note that only final cell numbers were measured for the bioreactor expanded cells; the curve represents exponential approximations of the previous days based on the overall growth rate from the experiment. (D) Predicted average cell density at different flow rates in a gas permeable wall packed bed bioreactor. The modelling predicts that the cell yield is almost independent of the flow rate.

When the model was modified to evaluate the effect of increased wall thickness, the wall offered little resistance to the diffusive flux of oxygen (S6 File) and had little overall effect on the cell density (Fig 3A). When the base model was adjusted to represent a traditional packed bed bioreactor (Fig 3B) by removing the oxygen equation for the wall expression (16) and replacing the boundary condition Eqs (17–20) with the boundary condition of no oxygen flux through the wall, that is

$$\frac{\partial C_{O,A}}{\partial r}=0\;\mathrm{at}\;r=R_{1},\;0<z<L,$$ (36)

the flow rate used in the oxygen-decoupled packed bed bioreactor experiments (0.208 ml/hr) was inadequate to support cell growth in a traditional packed bed bioreactor. A flow rate of 1.5 ml/min was required to achieve a similar cell growth as in the base model for the oxygen-decoupled packed bed bioreactor (Fig 3B).

**Fig 4:**
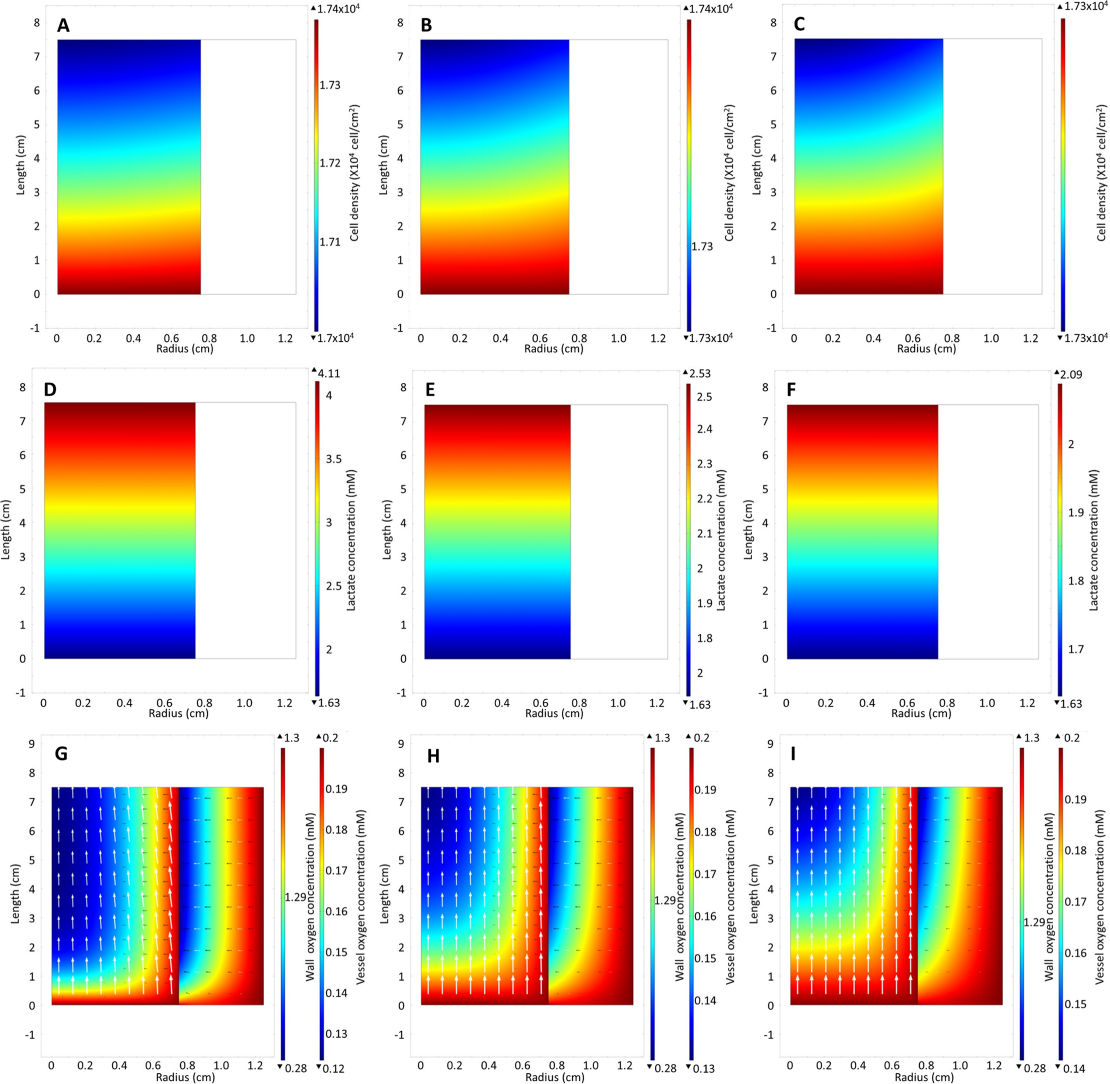
Heat maps of (A-C) cell density distribution (D-F) lactate concentration (G-I) and oxygen concentration at inlet flow rates0.5, 1.5, 3 cm^3^/hr respectively.

The base model predictions agree well with the experimental observations for cell expansion in the traditional T175 flask but agree poorly with the observations in the oxygen-decoupled packed bed bioreactor (Fig 3C). The base model was then modified to explore why the bioreactor cell growth was significantly less than traditional flask expanded cells. Increasing the model flow rate to 0.5, 1, 1.5, 3 and 4 ml/hr resulted in only a small increase in the average cell density (Fig 3D). Although the cells became more evenly distributed (Fig 4A–4C) as a result of the improved removal of lactate (Fig 4D–4F) and the increased supply of oxygen (Fig 4G–4I), this relative insensitivity to changes in flow rate suggests that the experimental flow rate used (0.208 ml/hr) was adequate for nutrient supply. At all flow rates, the shear stress acting on the cells was also significantly lower than the 0.015 Pa that has been shown to cause osteogenic differentiation of MSCs (S7 File) [7–9, 11].

**Fig 5:**
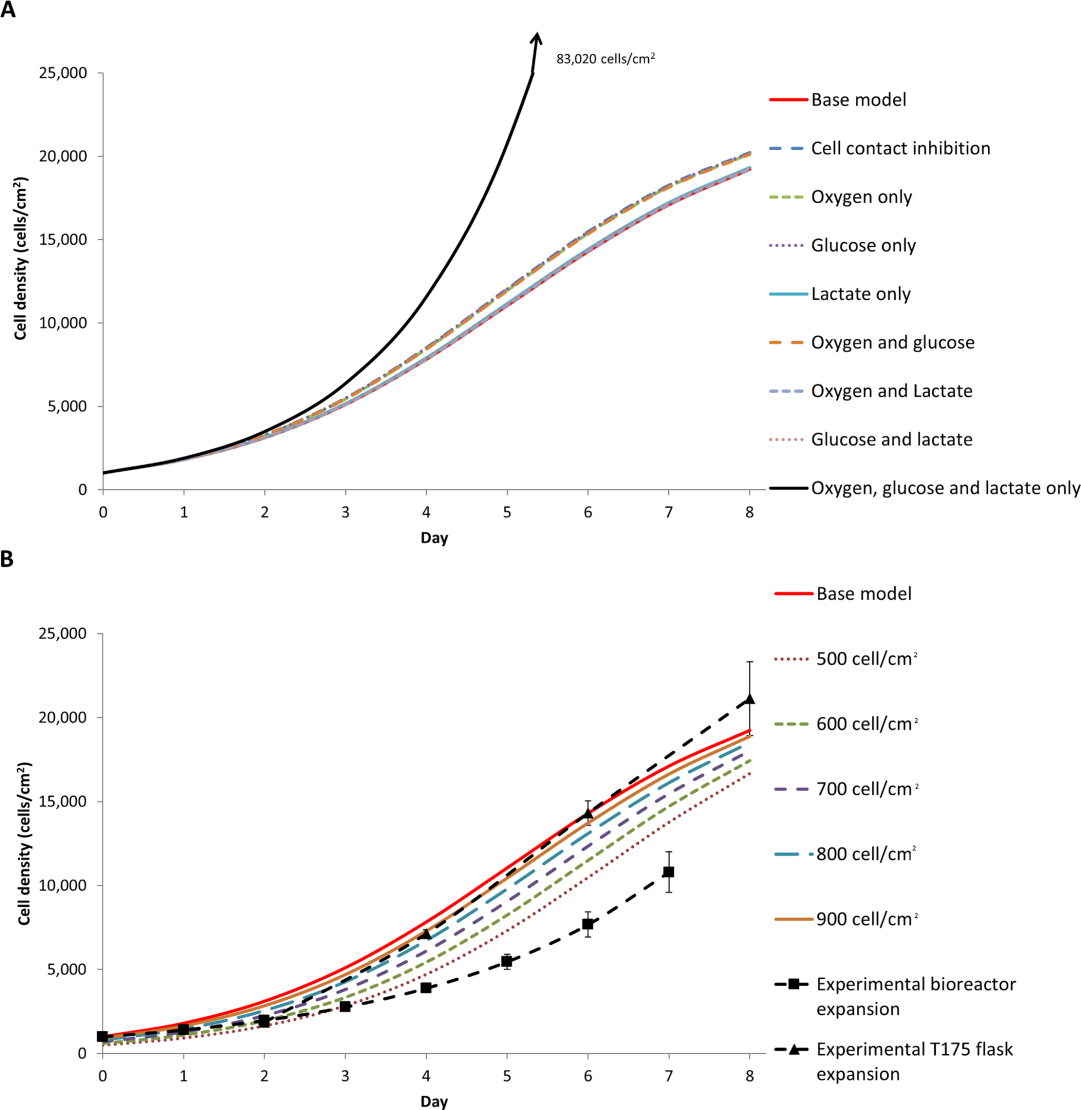
(A) The average cell density with different cell growth rate equations defined in Table 2. (B) The average cell density with the initial homogeneous cell distribution adjusted from 500 cell/cm^2^ to 1000 cell/cm^2^ (base model).

To evaluate if the 0.208 ml/hr flow rate used experimentally in the bioreactor was indeed adequate, and to determine which nutrient or waste metabolite was limiting the growth rate, the intrinsic growth rate in the cell balance expression (24) was modified. This modification explored the average cell density dependence on oxygen, glucose and lactate, as well as the cell contact inhibition, alone and in combination (Table 2). Other than cell contact inhibition, lactate showed the largest effect in reducing cell growth (Fig 5A), suggesting that lactate is the main driving parameter in developing the cell gradient across the length of the reactor. Both glucose and oxygen were likely in sufficient supply in our oxygen-decoupled packed-bed bioreactor, and that the compromised removal of lactate due to the low flow rate was likely responsible for the retarded cell growth. The K_L_ value, which is the concentration of lactate required to reduce the growth rate by half, is only 26 times larger than the initial concentration of lactate, whereas the K_O_ is 200 times smaller than the initial oxygen concentration. We conclude that the final cell number is much more sensitive to changes in lactate concentration than it is to the changes in oxygen concentration. This is true even if the oxygen concentration is reduced by a factor of two below the value used in our oxygen-decoupled packed bed bioreactor.

**Table 2:** Cell growth rate equations adjusted to account for different limitations in nutrient concentrations and cell contact inhibition.

**Condition**	**Cell density equation**
Base case	$\frac{\partial N}{\partial t}=K_{max}(\frac{C_{G}}{K_{G}+C_{G}}\frac{C_{O,A}}{K_{O}+C_{O,A}}\frac{K_{L}}{K_{L}+C_{L}})N\;(1-\frac{N}{N_{max}})+D_{N}\nabla^{2}N$
Cell contact inhibition	$\frac{\partial N}{\partial t}=N\;\left(1-\frac{N}{N_{max}}\right)+D_{N}\nabla^{2}N$
Oxygen	$\frac{\partial N}{\partial t}=K_{max}(\frac{C_{O,A}}{K_{O}+C_{O,A}})N\;\left(1-\frac{N}{N_{max}}\right)+D_{N}\nabla^{2}N$
Glucose	$\frac{\partial N}{\partial t}=K_{max}(\frac{C_{G}}{K_{G}+C_{G}})N\;\left(1-\frac{N}{N_{max}}\right)+D_{N}\nabla^{2}N$
Lactate	$\frac{\partial N}{\partial t}=K_{max}(\frac{K_{L}}{K_{L}+C_{L}})N\;\left(1-\frac{N}{N_{max}}\right)+D_{N}\nabla^{2}N$
Oxygen and glucose	$\frac{\partial N}{\partial t}=K_{max}(\frac{C_{G}}{K_{G}+C_{G}}\frac{C_{O,A}}{K_{O}+C_{O,A}})N\;\left(1-\frac{N}{N_{max}}\right)+D_{N}\nabla^{2}N$
Oxygen and lactate	$\frac{\partial N}{\partial t}=K_{max}(\frac{C_{O,A}}{K_{O}+C_{O,A}}\frac{K_{L}}{K_{L}+C_{L}})N\;\left(1-\frac{N}{N_{max}}\right)+D_{N}\nabla^{2}N$
Glucose and lactate	$\frac{\partial N}{\partial t}=K_{max}(\frac{C_{G}}{K_{G}+C_{G}}\frac{K_{L}}{K_{L}+C_{L}})N\;\left(1-\frac{N}{N_{max}}\right)+D_{N}\nabla^{2}N$
Oxygen, glucose and lactate only	$\frac{\partial N}{\partial t}=K_{max}(\frac{C_{G}}{K_{G}+C_{G}}\frac{C_{O,A}}{K_{O}+C_{O,A}}\frac{K_{L}}{K_{L}+C_{L}})N+D_{N}\nabla^{2}N$

Our model suggests that the oxygen supply would need to be nearly depleted to strongly affect the MSC growth rate. This result must be conservatively interpreted in the context of MSC expansion, as oxygen sensitivity can vary between cell types. MSCs prefer anaerobic respiration, but other cell types that prefer aerobic respiration result in a far higher K_O_ [40]. Given that the lactate/oxygen yield ratio is Y_L/G_ ≈ 2 for most cell types and culture conditions, the removal of waste products is apparently more important for cell growth than the supply of nutrients [40, 44, 45]. Simply adding glucose to the medium without increasing the flow rate could lead to accumulation of lactate in the bioreactor, leading to concomitant diminishing of the cell growth rate. Nevertheless, these simulations suggested that nutrient limitations are only a minor influence in the reduction of the experimental growth rate observed in our oxygen-decoupled packed bed bioreactor. Therefore, other factors must also be considered when attempting to interpret the experimental cell growth characteristics observed in the oxygen-decoupled packed bed bioreactor.

### Investigating the effect of cell distribution on growth

It was assumed in this theoretical treatment that all the cells initially attach to the scaffold with a homogeneous distribution, but this is almost impossible to achieve in practice. The cell seeding method would more likely lead to a heterogeneous initial cell distribution. Although having fewer cells attached to the scaffold reduced the final cell number (Fig 5B), this was not experimentally significant (see S8 File). The cell seeding method strongly influenced the cell distribution heterogeneity and the cell viability [46].

To model a heterogeneous cell distribution, while still maintaining same total cell number, the initial cell distribution was assumed to linearly decrease in either the axial (*z*) or radial (*r*) direction by specifying either the inlet or centre cell density (N_in_) between 0 cell/cm^2^ to 2000 cell/cm^2^ (note that this is converted to a volume concentration by multiplying by the factor, *a*, for the simulation). This is to represent poor seeding efficiency, in which cells are trapped at the inlet or settle to the bioreactor wall during the rocker-roller seeding process. The linear equations representing this are as follows:

$$N_{iz}=N_{in}+\left(p-N_{in}\right)\frac{z}{L}\;\mathrm{at}\;0<r<R_{1}\;\mathrm{and}\;0<z<L,$$ (37)

for a z-direction linear cell distribution and

$$N_{ir}=N_{in}+\left(pp-N_{in}\right)\frac{r}{R_{1}}\;\mathrm{at}\;0<r<R_{1}\;\mathrm{and}\;0<z<L,$$ (38)

for the r-direction linear cell distribution.

For both cell distributions, values of *p* and *pp* must be found that ensure the total cell number, T, remains fixed at about 158,800 cells. Expression (37) needed to be integrated between z = 0 cm to z = L for *πR*_*1*_^*2*^*N*_*iz*_ *= T*, therefore

$$\frac{T}{\pi R_{1}^{2}}=\int_{0}^{L}[N_{in}+\left(p-N_{in}\right)\frac{z}{L}]\;dz,$$ (39)

or

$$p=\frac{2T}{\pi R_{1}^{2}L}-N_{in}.$$ (40)

To solve for *pp*, expression (38) must be integrated between *r* = 0 and *r* = *R*_*1*_ with 2*πR*_*1*_*LN*_*iz*_ *= T*, therefore

$$\frac{T}{2\pi R_{1}L}=\int_{0}^{R_{1}}N_{in}+\left(pp-N_{in}\right)\frac{r}{R_{1}}dz,$$ (41)

or

$$pp=3\;\left(\frac{T}{2\pi R_{1}^{2}l}-\frac{N_{in}}{6}\right)\;\mathrm{at}\;0<r<R_{1}\;\mathrm{and}\;0<z<L.$$ (42)

**Fig 6:**
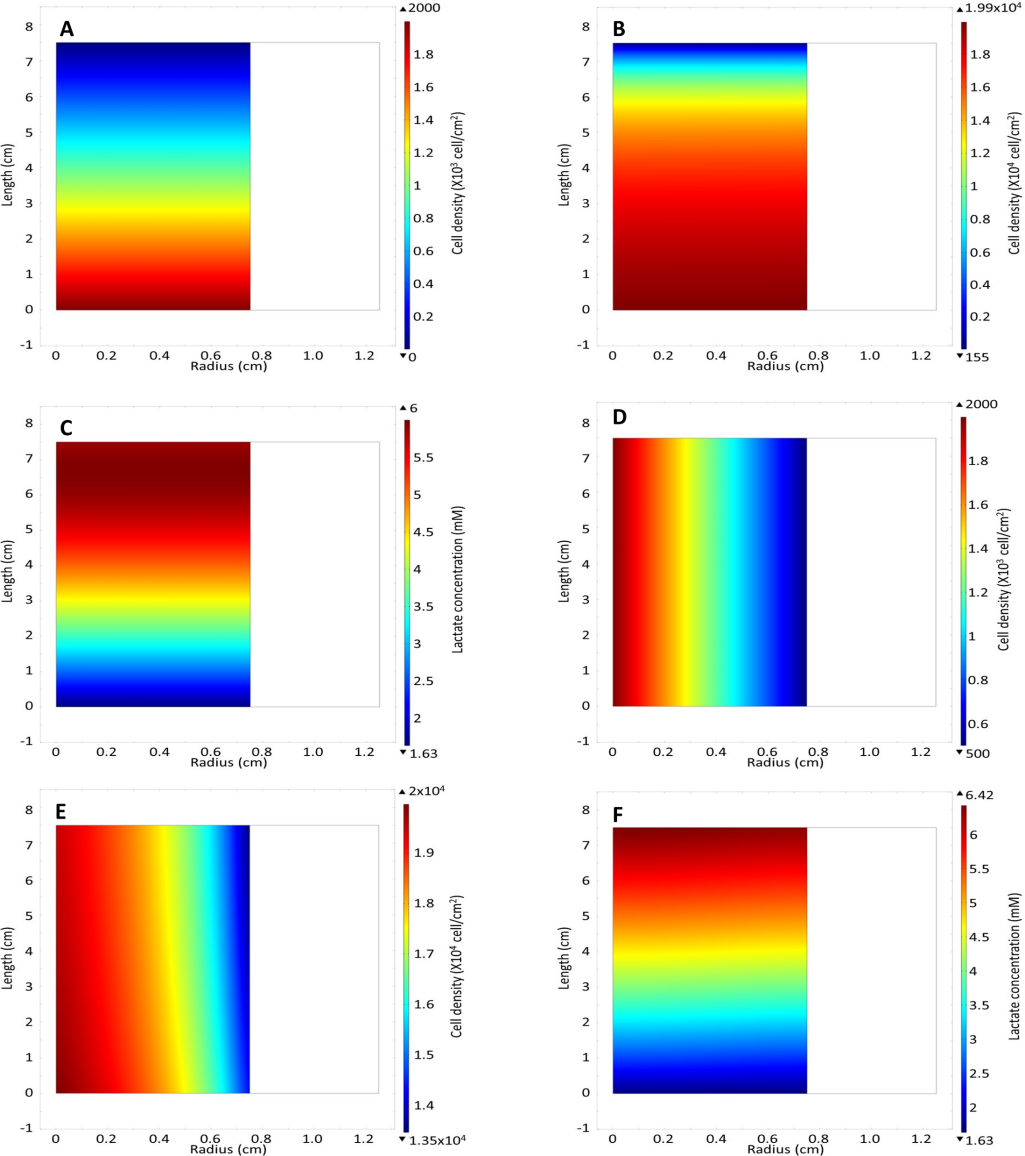
(A) Cell density heat map of linear cell distribution in the z direction at day 0 and with an inlet concentration of 2000 cell/cm^2^. (B) Cell distribution at day 7 with N_in_ = 2000 cell/cm^2^ with a linear cell distribution in the z direction. (C) Lactate concentration at day 7 with a N_in_ = 2000 cell/cm^2^ with a linear cell distribution in the z direction. (D) The initial cell density of linear cell distribution in the r direction with a centre concentration of 2000 cell/cm^2^. (E) Cell density of linear cell distribution in the r direction at day 7 with N_in_ = 2000 cell/cm^2^. (F) Lactate concentration for linear cell distribution in the r direction with N_in_ = 2000 cell/cm^2^ at day 7.

The initial cell distributions described by (37) and (38) are shown in Fig 6A & 6D, respectively.

**Fig 7:**
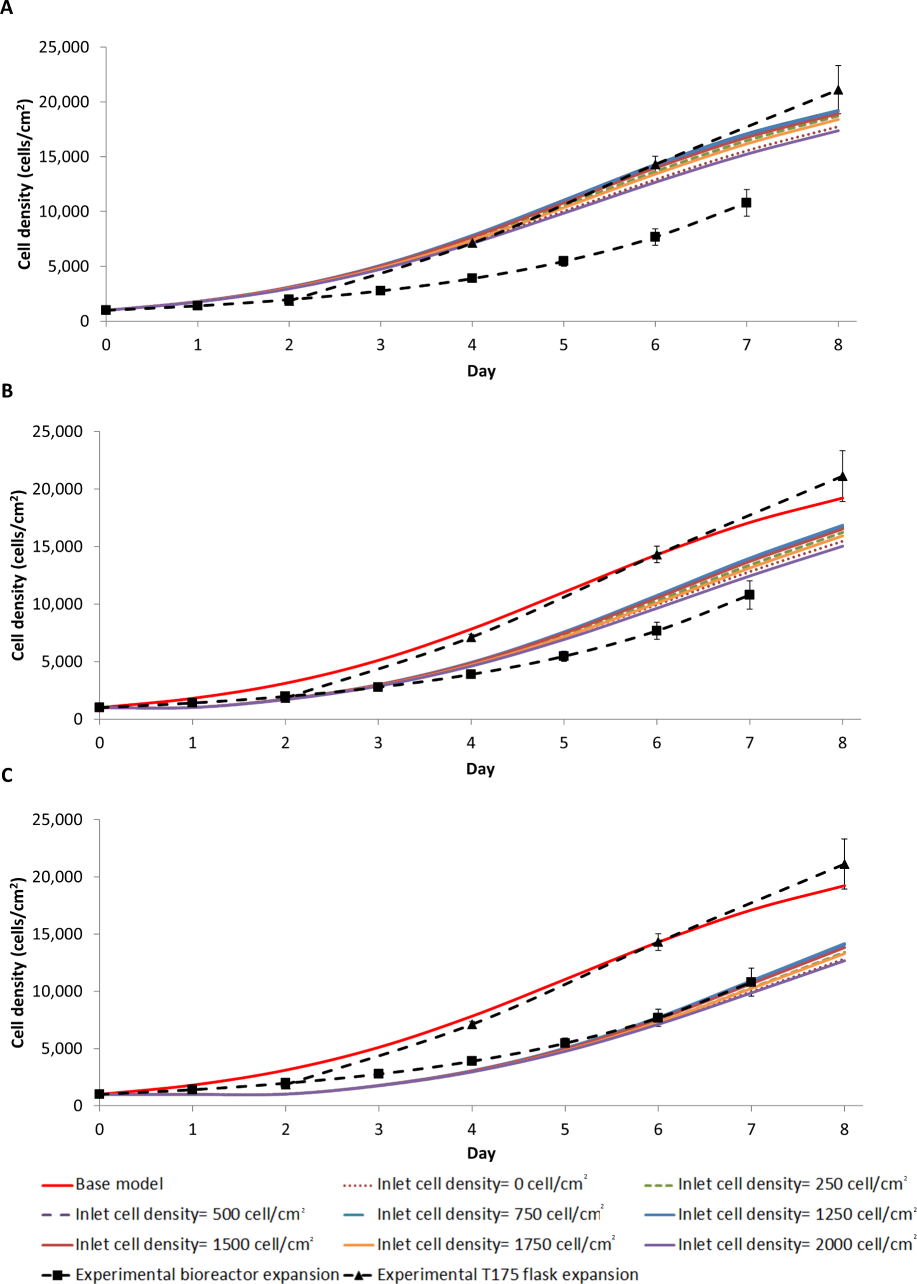
Heterogeneous cell distributions are modelled as a linear function in the z direction, setting the cell concentration at the inlet (z = 0 cm) between 0 to 2000 cell/cm^2^with (A) 0 hr, (B) 24 hr and (C) 48 hr cell quiescence. Note that the total initial cell number is the same in all conditions (158,800 cells).

**Fig 8:**
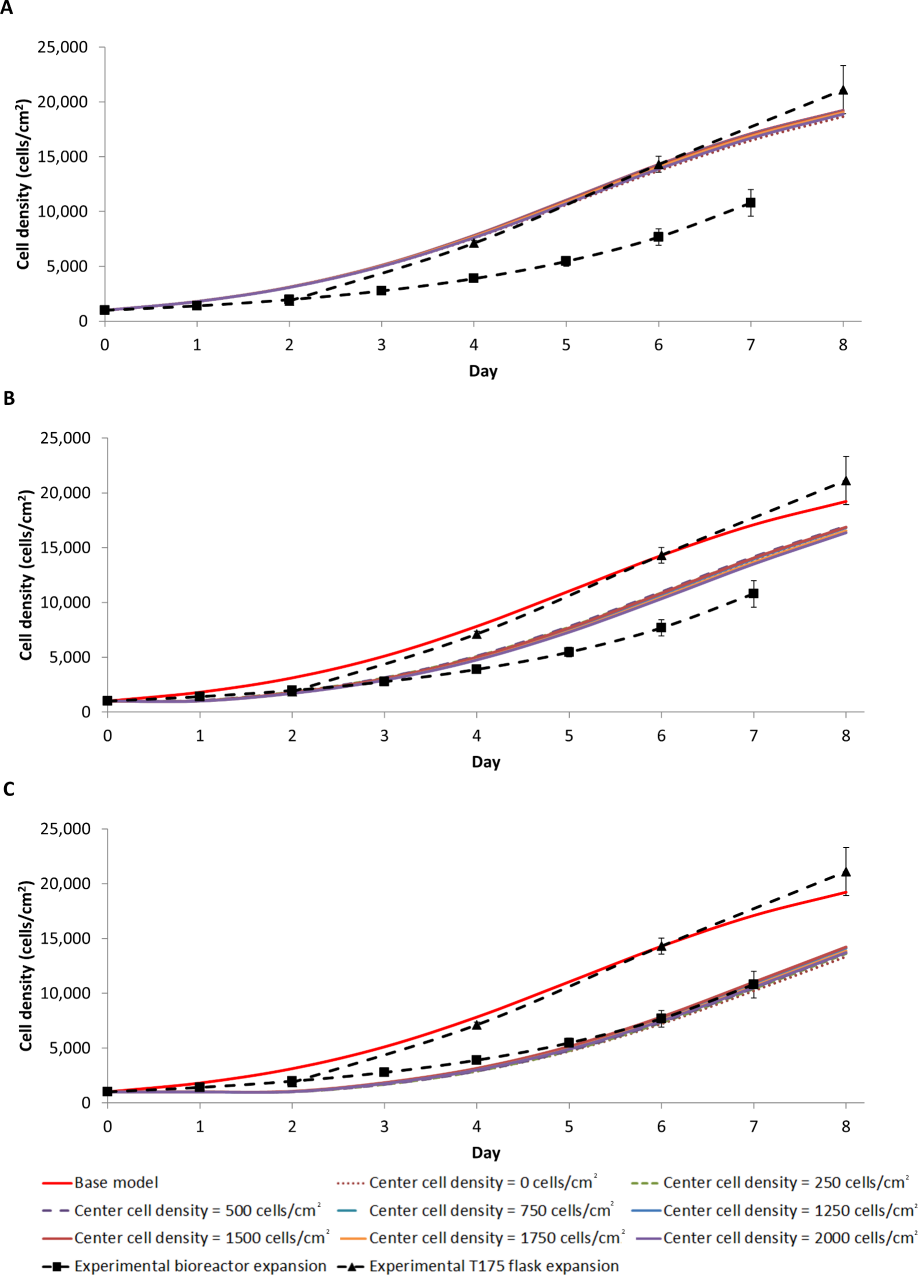
Heterogeneous cell distribution modelled as a linear function in the r direction by setting the centre cell density (r = 0) between 0 to 2000 cell/cm^2^ with 0 hr (A), 24 hr (B) and 48 hr (C) cell quiescence. Note that the total initial cell number is the same in all conditions (158,800 cells).

A linear heterogeneous cell distribution strongly impacts the final average cell density, and does so more in the z-direction than in the r-direction (Fig 7A & Fig 8A). The addition of a heterogeneous cell distribution also resulted in a better fit to the experimental data, with heterogeneous cell distribution largest RMSE in Table 3 is 4107 cell/cm^2^ compared to the base model RMSE of 4133 cell/cm^2^. The cell density heat maps show that a larger final cell distribution gradient develops in the z-direction. The cell density in the inlet half of the bioreactor reaches near confluence, while the outlet half reaches a cell density of 114 cell/cm^2^ (Fig 6B). Conversely, the cell density gradient is reduced with an initial non-uniform linear cell distribution in the r direction, with a difference of 2x10^4^ cells/cm^2^ in the centre of the bioreactor compared to 1.35x10^4^ cells/cm^2^ at the reactor wall (Fig 6E). This difference is firstly due to the smaller initial distribution in the r direction than in the z-direction (Fig 6A & 6D). However, lactate build-up inhibiting the cell growth is the main driver of a large cell density gradient, which is more significant when the initial cell distribution in the z-direction is non-uniform. Although the lactate concentration does not reach as high a level in z-direction heterogeneous seeding, a larger volume of the bioreactor is exposed to a higher lactate concentration (Fig 6C & 6F), resulting in a reduced growth rate for cells located closer to the outlet. When this effect was modelled in the heterogeneous initial cell distribution in the r direction, a cell density gradient developed along the length of the reactor (Fig 6E). We concluded that a heterogeneous initial cell distribution alone is insufficient to cause the significant difference that was observed experimentally between the cell yield in the bioreactor expansion and the traditional flask culture (Table 3).

**Table 3:** The root-mean-square error (RMSE) for effect of cell distribution and cell lag on the model fit to experimental data (cells/cm^2^).

**Centre cell density (cells/cm^2^)**	**0**	**250**	**500**	**750**	**1250**	**1500**	**1750**	**2000**
**RMSE radial, no lag**	3772	3946	4047	4102	4107	4062	3987	3878
**RMSE radial, 24 hr lag**	1706	1845	1900	1759	1767	1831	1583	1500
**RMSE radial, 48 hr Lag**	924	847	816	670	658	620	734	774
**Inlet cell density (cells/cm^2^)**	**0**	**250**	**500**	**750**	**1250**	**1500**	**1750**	**2000**
**RMSE axial, no lag**	3236	3728	3980	4102	4075	3929	3565	3062
**RMSE axial, 24 hr lag**	1219	1460	1658	1759	1739	1618	1320	1030
**RMSE axial, 48 hr lag**	1031	874	708	664	672	726	898	1102

**Fig 9:**
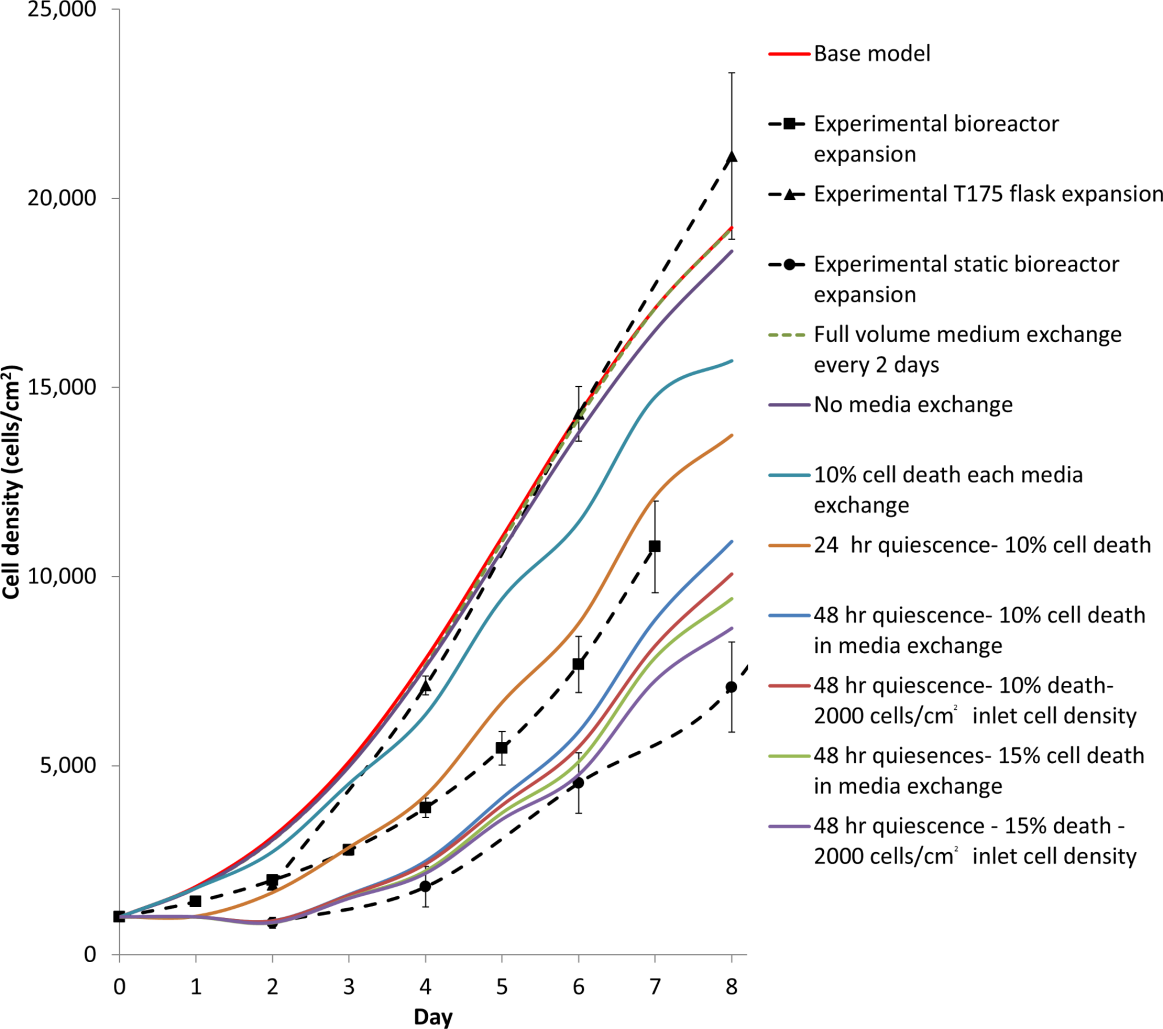
Experimental and model average cell densities of a batch bioreactor with a full volume exchange every two days, exploring the effect of no medium exchange, cell death with every medium exchange, quiescence due to cell shock and heterogeneous cell distribution.

However, 24 to 48 hour delay in the cell growth was consistently observed in all experimental expansions, consistent with literature reports [47–49]. The quantified cell density measured on day two illustrates this lag in the experimental static bioreactor expansion growth curve (Fig 9). We posit that this lag represents a phase in which the cells recover from extra handling and stress during the seeding process. The effect of a lag phase on cell proliferation was evaluated by introducing two lag phase time points (24 hr and 48 hr). During the perspective lag phases the cells could migrate but not proliferate. The growth term in Eq (25) was modified as follows:

$$k=H[t-T]\;\left(K_{max}(\frac{C_{G}}{K_{G}+C_{G}}\frac{C_{O,A}}{K_{O}+C_{O,A}}\frac{K_{L}}{K_{L}+C_{L}})\right).$$ (43)

Here H[t] is the Heaviside function and is given by

$$H[t]=\left\{ \begin{array}{c} 0,t<T\\ 1,t\geq T. \end{array}\right.$$ (44)

When the initial delay in proliferation is 24 hours we take T = 24 and when the initial delay is 48 hours we take T = 48.

The modified model better fit the experimental oxygen-decoupled packed bed bioreactor growth data (Fig 7B & 7C, Fig 8B & 8C & Table 3), suggesting that a combination of a non-uniform initial cell distribution in the axial direction and an initial time lag in cell proliferation may explain the observed difference in cell yield between the bioreactor and the traditional flask culture. Thus, distributing cells uniformly during the seeding process while minimising stress to the cells is the most important factor when optimising efficient cell expansion in a 3-dimensional culture device such as a packed bed bioreactor. While many bioreactor seeding methods have been reported [46, 50–53], inhomogeneity and cell stress have greatly affected the overall cell expansion in each, suggesting that this is very difficult to achieve in practice.

### Modelling a batch bioreactor

The model was modified to a batch reactor configuration to investigate whether the cell distribution and cell growth lag were the main factors in bioreactor reduced growth rate compared to a flask culture. To do this, the terms involving the fluid flow through the scaffold were removed, and the mass balance equations for glucose (6), lactate (7) and oxygen (15) were modified as follows,

$$\frac{{\partial C}_{G}}{\partial t}=\nabla .(D_{G}\nabla C_{G})-\frac{V_{max,G}NC_{G}}{K_{m,G}+C_{G}},$$ (45)

and

$$\frac{{\partial C}_{L}}{\partial t}=\nabla .(D_{L}\nabla C_{L})+Y_{L/C}\frac{V_{max,G}NC_{G}}{K_{m,G}+C_{G}},$$ (46)

and

$$\frac{\partial C_{O,A}}{\partial t}=\nabla .\left(D_{O}\nabla C_{O,A}\right)-\frac{V_{max,O}NC_{O,A}}{K_{m,O}+C_{O},A}\;\mathrm{for}\;0<r<R_{1}.$$ (47)

The source boundary conditions Eqs (11) and (21) were also removed, with the addition of no flux boundary condition at the inlet for glucose, lactate and oxygen, defined as

$$\frac{\partial C_{i}}{\partial z}=0\;\mathrm{at}\;z=0,\;0<r<R_{1}.$$ (48)

In the experimental protocol, a full volume medium exchange was performed every two days. To model this, the simulation was run for 48 hours. The cell density output was used as the initial cell distribution for the next simulation of 48 hours. This was repeated for the required 8 days of cell growth.

Even without medium exchanges, the cell growth predicted in a batch reactor system was close to the predicted cell growth for the oxygen-decoupled packed bed perfusion bioreactor (Fig 9). However, the experimental batch bioreactor results were significantly less than the experimental perfusion results [12]. As the protocol for measuring the cell number every two days required multiple full-volume medium exchanges to infer the cell numbers, the resulting stress might have killed or detached and washed away some of the cells. To explore this hypothesis, the model was modified so that the cell density was reduced by between 10% and 15% when the output cell density from the previous time period was used as the initial value. A heterogeneous initial cell distribution and lag phase was also added so the model achieved a better fit to the static bioreactor expansion experimental results (Table 4). This model was consistent with killing or detaching a proportion of the cells after each medium exchange during the protocol to measure the cell numbers, and confirmed that heterogeneous cell distribution and a lag phase are the main driving factors of the reduced growth in the oxygen-decoupled packed bed bioreactor. These data suggested a change in protocol for the oxygen-decoupled packed bed bioreactor expansion to perform cell quantification only at the end of the culture period.

**Table 4:** RMSE for different model scenarios fit to batch reactor experimental data.

Scenario	RMSE (cell/cm2)
**Base Case**	8430
**Full volume medium exchanges every 2 days**	8352
**No media exchange**	8019
**10% cell death each medium exchange**	6050
**24 hr quiescence- 10% cell death**	4143
**48 hr quiescence- 10% cell death in medium exchange**	2072
**48 hr quiescence- 10% death- 2000 cells/cm2 inlet cell density**	1600
**48 hr quiescence- 15% cell death in medium exchange**	1221
**48 hr quiescence—15% death—2000 cells/cm2 inlet cell density**	804

### Using the model to investigate scaling up

A packed bed bioreactor can be scaled-up in two ways, by either increasing the dimensions of the reactor itself, or by reducing the size of individual particle to increase the surface area. Experiments were carried out on a scaled-up oxygen-decoupled packed bed bioreactor to increase the reactor volume while retaining the same pellet size [12]. However, equipment limitations prevented us from optimising the flow rate. A cyclic perfusion system was employed, rather than the preferred single pass circuit, and the lowest flow rate available was 30 ml/hr. To direct further scale up modifications and their optimisation, simulations were performed using the model that was developed.

The geometry of the model was changed to represent the oxygen-decoupled packed bed bioreactor used in the trial scale-up experiments. The radius was adjusted so that the scaffold radius was R_1_ = 2.5 cm while the wall thickness remained at 0.5 cm, therefore R_2_ = 3 cm. The length was increased to L = 12 cm, which increased the reactor volume to 235.6 cm^2^. With the same pellet size as the base model, this increased the reactor surface area to 2830 cm^2^. Assuming a cell density of 20,000 cells/cm^2^, this surface area is sufficient to support 5.66x10^7^ cells.

**Fig 10:**
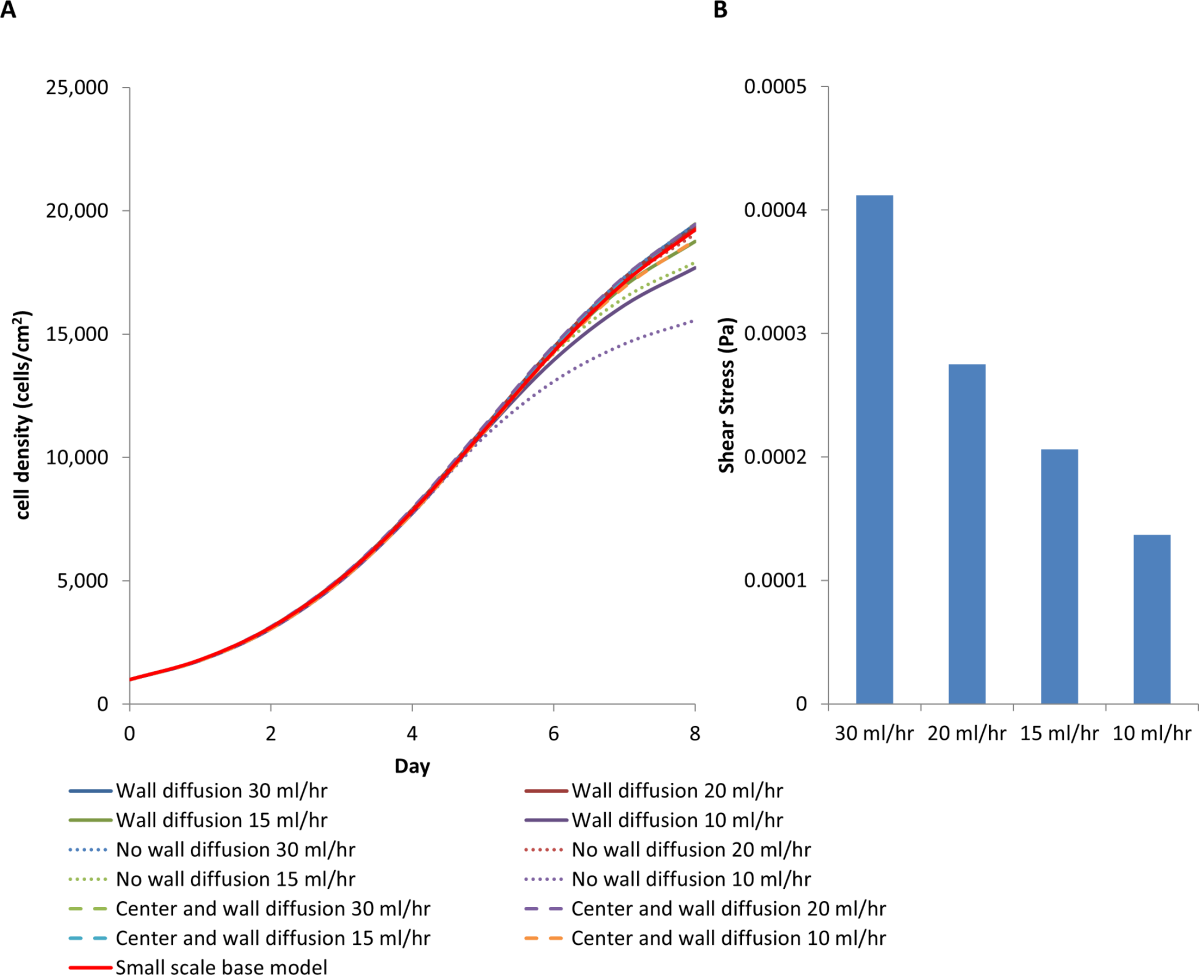
(A) Average cell density comparing scaled-up bioreactor R_1_ = 2.5 cm, R_2_ = 3 cm and L = 12 cm with bioreactor design that includes wall diffusion, no wall diffusion, wall diffusion with additional central capillary providing oxygen. (B) Shear stress at different flow rates.

**Fig 11:**
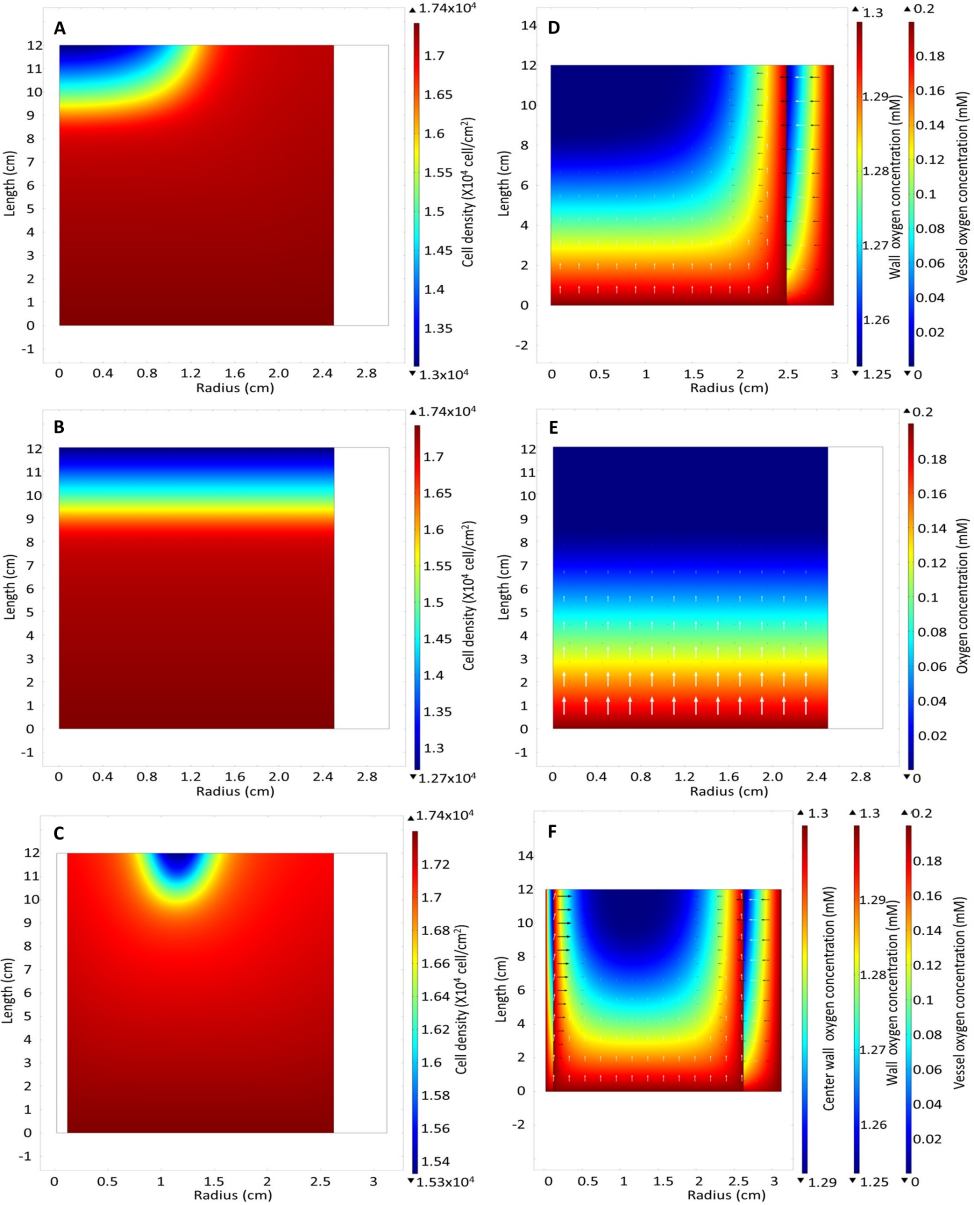
Cell density heat map of bioreactor with (A) oxygen provided by diffusion through the wall, (B) no wall diffusion and (C) oxygen provided by both diffusion through the wall and central capillary at day 7 at a flow rate 15 ml/hr. Oxygen concentration with (D) oxygen provided by diffusion through the wall, (E) no wall diffusion and (F) oxygen provided by both diffusion through the wall and central capillary at day 7.

The average cell density achieved in the scaled-up oxygen-decoupled packed bed bioreactor was similar to that obtained in the base model with a medium perfusion rate of 15 ml/hr (Fig 10A). The shear stress at all flow rates was significantly below the 0.015 Pa that has been shown to induce osteogenic differentiation (Fig 10B). However, it was apparent that the radial oxygen diffusion may not be sufficient to support cell growth at a perfusion rate below 15 ml/hr, with the oxygen concentration nearing depletion (Fig 11A & 11D). This suggests that additional oxygen sources would be required if the diffusion length is greater than 1 cm. Nevertheless, the design superiority of the wall diffusion bioreactor over the traditional packed bed bioreactor with no oxygen sources other than through medium perfusion is even more apparent in the scaled-up bioreactor. A decrease in the average cell density was observed with medium perfusion flow rates below 20 ml/hr (Fig 10A).

In the traditional packed-bed bioreactor at flow rates of 15 ml/hr, an increasing region of hypoxia is predicted at the bioreactor outlet that results in a decreased cell density at the outlet (Fig 11B & 11E). At the higher flow rates required for the scale up model, the lactate is removed at a sufficiently rapid rate to ensure that its concentration does not significantly affect cell growth (S9 File).

To counteract this oxygen depletion and allow for lower flow rates, the base model was extended to include additional oxygen sources in the form of capillaries running through the bioreactor. Due to the limitations inherent in the axisymmetric model, only one capillary could be added at the centre of the bioreactor. In the geometry outlined in (Fig 1D), the lumen radius, R_c_, is 0.02 cm with the capillary wall outer radius, R_w_, is 0.12 cm, the vessel radius, R_1_, is 2.62 cm and the outer wall, R_2_, is 3.12 cm, the reactor length L = 12 cm. In this model, the capillary wall is composed of polymer with the same gas permeability as PDMS. The boundary conditions for the oxygen transport within the vessel were modified to account for oxygen diffusion through the central wall. Therefore, the concentration of oxygen within the capillary wall (C_O,C_) is given by the solution of the equation

$$\frac{\partial C_{O,C}}{\partial t}=D_{PDMS}\nabla^{2}C_{O,C}\;\mathrm{for}\;R_{c}<r<R_{w}.$$ (49)

The same boundary conditions apply at the outer vessel wall and for the capillary. There was zero flux of oxygen through the inlet and the outlet.

$$\frac{\partial C_{O,B}}{\partial z}=0\;\mathrm{at}\;z=0,\;L,\;R_{c}<r<R_{w,}$$ (50)

A constant oxygen concentration at the capillary lumen wall was assumed.

$$C_{O,C}=S_{PDMS}\;\mathrm{at}\;r=R_{c},0<z<L.$$ (51)

At the interface between the capillary wall and the medium, the boundary condition Eq (23) is now replaced by

$$D_{O}\frac{\partial C_{O,A}}{\partial r}=D_{PDMS}\frac{\partial C_{O,C}}{\partial r}\;\mathrm{at}\;r=R_{w},\;0<z<L$$ (52)

And

$$\frac{C_{O,A}}{S_{M}}=\frac{C_{O,C}}{S_{PDMS}}\;\mathrm{at}\;r=R_{w},\;0<z<L.$$ (53)

This design change to the oxygen-decoupled packed bed bioreactor model predicted superior cell expansion at lower flow rates, allowing the medium to be perfused at 10 ml/hr and resulting in a similar growth rate to the base model (Fig 10A). No depletion of oxygen at the outlet of the bioreactor was predicted (Fig 11F). However, the oxygen concentration is 10-fold less than the Michaelis-Menten oxygen growth constant K_O_, resulting in a slower growth rate at r = 1.25 cm in the bioreactor (Fig 11C). Further modelling analyses using 3-dimensional geometric models to allow for the addition of more oxygen-providing capillaries must be employed to determine the optimal distance between capillaries.

A decrease in the particle size of the pellets further increases the available surface area. By decreasing the cylindrical pellet size to 1mm diameter by 1mm in length the reactor surface area increases to 7,493 cm^2^, which is sufficient to support 1.5x10^8^ MSCs. Further decreasing the cylinder pellet size to 0.5 mm diameter by 0.5 mm in length increases the surface area to 14,985 cm^2^, which can support 3x10^8^ MSCs. The hypothetical improvement in yield can be evaluated by modifying the current model.

**Fig 12:**
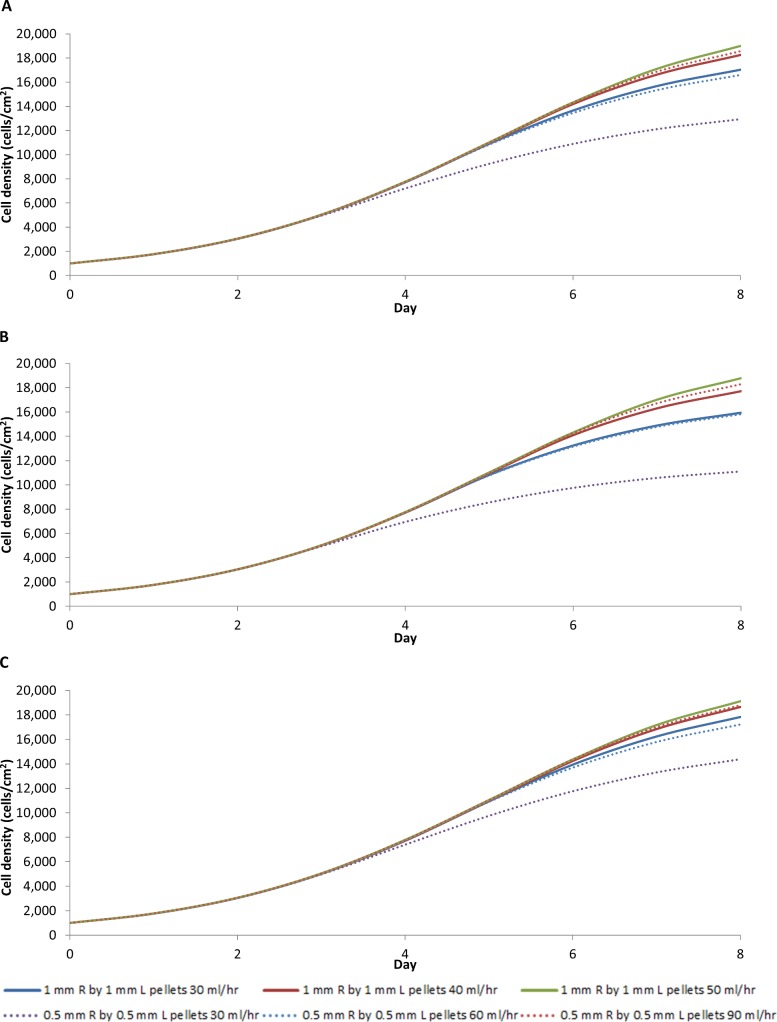
Average cell density in the larger R_1_ = 2.5 and L = 12 cm bioreactor with different pellet and flow rate for a bioreactor design that includes wall diffusion (A), no wall diffusion (B), wall diffusion with additional central capillary providing oxygen (C).

**Fig 13:**
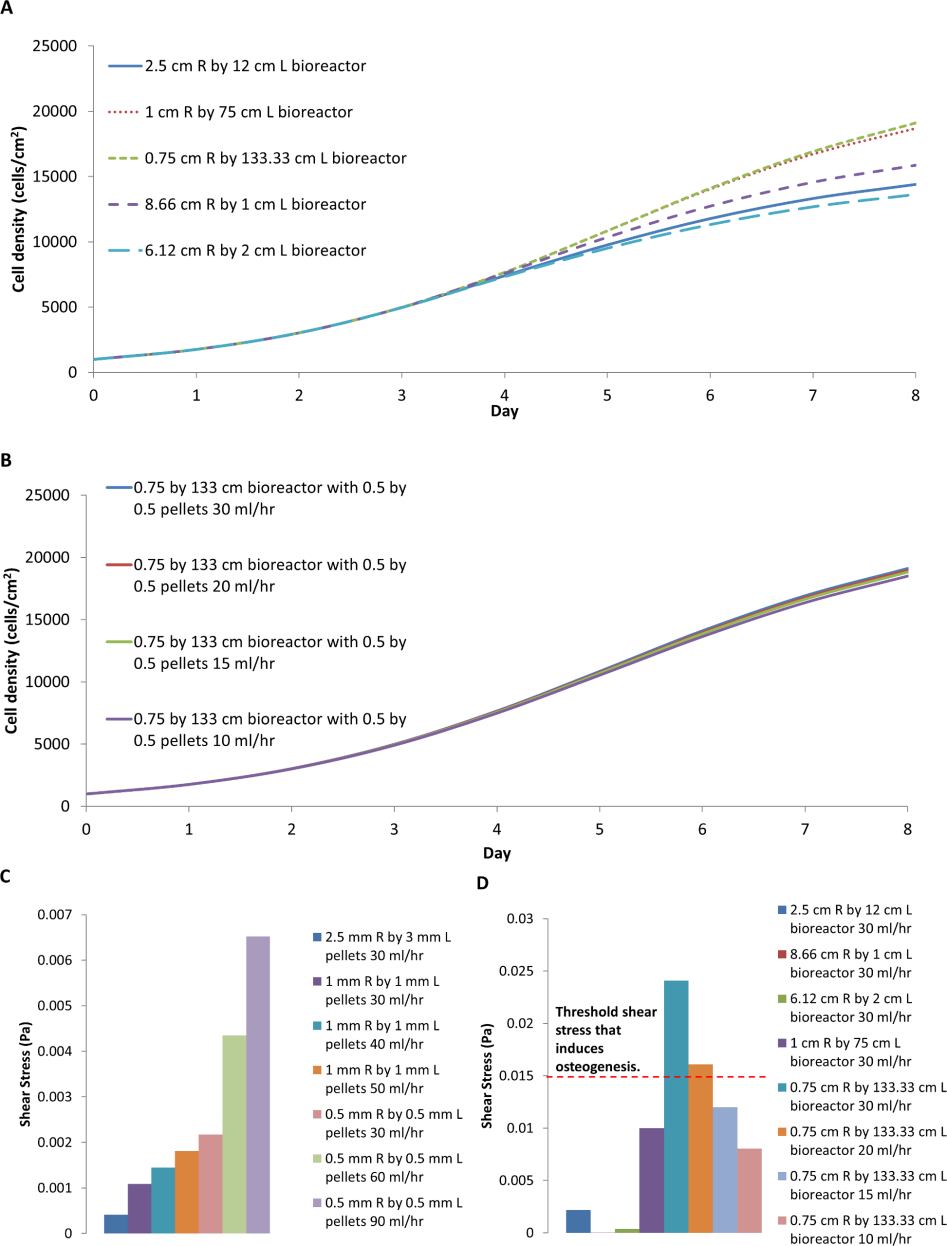
(A) Average cell density of the bioreactor design that provides oxygen by diffusion through the wall and central capillary using 0.5 mm pellets with different bioreactor geometries with the same reactor volume and surface area as a R_1_ = 2.5 and L = 12 cm bioreactor with a flow rate of 30 ml/hr. (B) Average cell density of the bioreactor providing oxygen through the wall and central capillary at different flow rate rates. (C) Shear stresses of different pellet size and different flow rates. (D) Shear stresses of different bioreactor geometries and flow rate.

The simulations show that the average cell density is significantly less with both smaller pellet bioreactor designs, with or without wall diffusion, unless the flow rate is increased to 50 ml/hr for the 1mm by 1mm pellets when the average cell density reaches 19000 cells/cm^2^ (Fig 12A & 12B). Neither design, with or without wall diffusion, supported a pellet size of 0.5mm by 0.5mm because oxygen would become depleted without a significant increase in flow rate. An increased flow rate can potentially be employed, as a flow rate of 90 ml/hr would still yield a shear stress below the 0.015 Pa threshold (Fig 13C). However, such a high flow rate would require an increase in medium, which would increase the cost. A decrease in flow rate would require adding a central capillary to provide sufficient oxygen to allow optimal growth rate (Fig 12C). Any further decrease the flow rate would require additional oxygen supply beyond the single central capillary.

Changing the reactor shape is an alternative to increasing the number of capillaries providing oxygen into the reactor and reduce the flow rate. To explore this alternative, the model reactor dimensions were modified without changing the reactor volume. Fig 13A shows the average cell density bioreactor with oxygen supplied through the wall and centre maintaining a reactor volume of 235.6 cm^3^ filled with 0.5 mm by 0.5mm cylindrical pellets changing the geometry of the reactor by adjusting the radius, R_1_, and the length, L, while still maintaining a perfusion rate at 30 ml/hr. A long thin bioreactor performs the best in our model, while a short fat reactor requires a higher flow rate to achieve the same growth rate, because oxygen is better capable of diffusing through to the centre of the long thin vessel. Our model suggests that the maximum distance between oxygen sources should be between 0.75 cm and 1 cm. The flow rate can to be reduced in this way without significantly affecting the cell growth rate (Fig 13B). Since the shear stress for the long thin reactor is only above the 0.015 Pa threshold when the flow rate is above 15 ml/hr, this shape change provides a suitable alternative reactor design (Fig 13D).

Further improvements to the model to enhance the design for scale up should be considered in the future. It was assumed that the cellular growth and extra cellular matrix production does not affect the pore size, because the pore size of the bioreactor scaffold is approximately 1000-fold larger than the size of the cells and their potential extracellular matrix production. As part of future scale up, in order to increase surface area, the particle size may be reduced, this will influence the pore size. Therefore, the model should be improved to include the effect of the cellular monolayer growth and extra cellular matrix production of MSCs on the pore size, as previously been modelled by Chung *et al*. [25]. This step is necessary to investigate the pore size reduction on the fluid flow and transport of nutrients and waste products. Furthermore, a full three-dimensional model should be developed to explore the capillary layout that could support an adequate cell number for the large scale expansion of MSCs for clinical use.

## Conclusion

A novel 2-dimensional axisymmetric model was developed to explore a packed bed bioreactor that provided an additional source of oxygen by diffusion through the reactor wall. This model accounts for the effects of oxygen, glucose and lactate on the cell growth using Michaelis-Menten kinetics. The fluid flow through the scaffold is assumed to follow Darcy’s Law.

The model developed here was applied to investigate our previous experimental observation that the oxygen-decoupled packed bed bioreactor expanded cells exhibited a reduced growth rate compared to cells grown in traditional tissue culture flasks. Traditionally, bioreactor device development/design focusses on optimising the supply of oxygen and glucose [17–19]. Our small-scale bioreactor model suggested that the difference in cell growth rate was not due to poor nutrient supply and that oxygen diffusion through the gas permeable wall was adequate to promote cell growth. However, accumulated lactate concentration was predicted to impact the cell growth rate more strongly than the nutrient levels of oxygen and glucose. Given the conversion ratio of glucose to lactate in most cell types, Y_L/G_ ≈ 2, the accumulation of lactate is significantly greater than the consumption of glucose [40, 44, 45]. In addition, the model suggests that the Michaelis-Menten growth constant plays an important role in the cells’ increasing sensitivity to lactate. Thus, greater consideration should be given the removal of waste metabolites in bioreactor designs, especially in low flow rate devices.

Compared to a traditional packed-bed bioreactor, our packed-bed bioreactor with gas-permeable walls decouples the oxygen supply from the bulk nutrient supply in the medium, reducing the perfusion flow rate by as much as a factor of five [12]. An initial heterogeneous cell distribution included in the mathematical model to help explain the experimental bioreactor data by assuming a linear cell distribution in either the z- or r-directions. In the z-direction cell distribution calculations, most cells to adhere to the scaffold near the inlet, resulting in a far greater lactate production due to the localised high cell density. The lactate increases at the inlet influences cell growth at the outlet. The model predicted a further reduction in the growth rate of the cells when a lag phase in the cell growth due to the stresses associated with the cell seeding process was included. This refined model yielded a simulation that closely matched the observed experiment results, suggesting that the cell seeding method is the most important factor to optimise, to yield the highest average cell density during bioreactor cell expansion. New seeding methods that achieve a more uniform cell distribution while limiting cell stress will be important in future bioreactor design.

A scaled-up bioreactor was modelled to direct future development of our gas permeable bioreactor design. When the reactor vessel volume was increased to 235.6 cm^3^, the resulting higher medium flow rates ensured that the lactate concentration never exceeded the threshold that would decrease the cell growth rate. Oxygen diffusion, however, became the limiting factor. To supply adequate oxygen to cells in the scaled-up bioreactor, a central gas permeable capillary was incorporated into the model. In addition, the particle size was decreased to increase the total growth surface area. Further changes were required to the bioreactor geometry to increase the cell number beyond 3x10^8^ cells. The mathematical model incorporating a central gas permeable capillary predicts that a R_1_ = 0.75 cm and L = 133 cm bioreactor can support this cell number at a low flow rate of 10 ml/hr. If additional capillaries are to be incorporated, the centrosymmetric model suggests that the maximum distance between additional capillaries can be no greater than 0.75cm as a first approximation. To validate this simple approximation, the model would need to be modified to a more complex full 3-dimensional geometry.

The mathematical modelling of this packed bed bioreactor with gas permeable walls supports the notion that decoupling the oxygen supply from the bulk nutrient supply represents a substantial improvement to the traditional packed bed devices. It allows for reduced medium flow rates, which decreases the cost of the cell expansion and potentially reduces shear stress on the cells. The mathematical model helped to interpret the our previous experimental results [12] by identifying the importance of the initial cell distribution on the final cell number. Further, the model directed future development and scale-up of the bioreactor design by suggesting the addition of diffusive oxygen sources.

## Supporting information

S1 File Mesh refinement study, difference in final average cell density (cell/cm^2^) at different mesh sizes (cm). (DOCX)

S2 File Average cell density of the base model. (DOCX)

S3 File Predicted oxygen concentration (mM) in reactor vessel and PDMS wall of the base model. (DOCX)

S4 File Predicted glucose concentration (mM) using the base model. (DOCX)

S5 File Predicted lactate concentration (mM) using the base model. (DOCX)

S6 File Oxygen concertation (mM) at the centre and wall interface with varying wall thickness at day 7. (DOCX)

S7 File Predicted shear stress as a function of medium flow rate. (DOCX)

S8 File Seeding efficiency of using the rocker-roller method compared to straightforward injection of the cells, allowing them to attach under static conditions, (n = 4, mean + SD). (DOCX)

S9 File Predicted glucose and oxygen concentrations for different flow rates in scaled up bioreactor model. (DOCX)
