# Supplementary material for: Modelling mesenchymal stromal cell growth in a packed bed bioreactor with a gas permeable wall
Source: PLoS One. 2018 Aug 27;13(8):e0202079. doi: 10.1371/journal.pone.0202079 (PMC6110476; doi:10.1371/journal.pone.0202079)
Supplement: S1 File — (DOCX) [file pone.0202079.s001.docx]

A mesh refinement study was performed using the base model to investigate the grid mesh dependence of the predictions bearing in mind that a very fine mesh implies a very large computation time. The maximum mesh size was reduced in steps from 0.1cm to 0.02 cm. The difference between the average cell density at a mesh size of 0.02 cm and the larger mesh sizes are plotted below. It was decided choosing a mesh fine enough to give predictions that were within 0.05 cell/cm^2^of the finest mesh was acceptable. Thus a maximum mesh size of 0.05 cm was adopted as it provides an acceptable tolerance and practical computing time.
