# Supplementary material for: Modelling mesenchymal stromal cell growth in a packed bed bioreactor with a gas permeable wall
Source: PLoS One. 2018 Aug 27;13(8):e0202079. doi: 10.1371/journal.pone.0202079 (PMC6110476; doi:10.1371/journal.pone.0202079)

Heat map of the cell density within the bioreactor calculated from the base model every two days. It shows the development of the non-uniform cell gradient from the inlet to the outlet which increases over time.

Cell density (x10^3^ cells/cm^2^) day 2


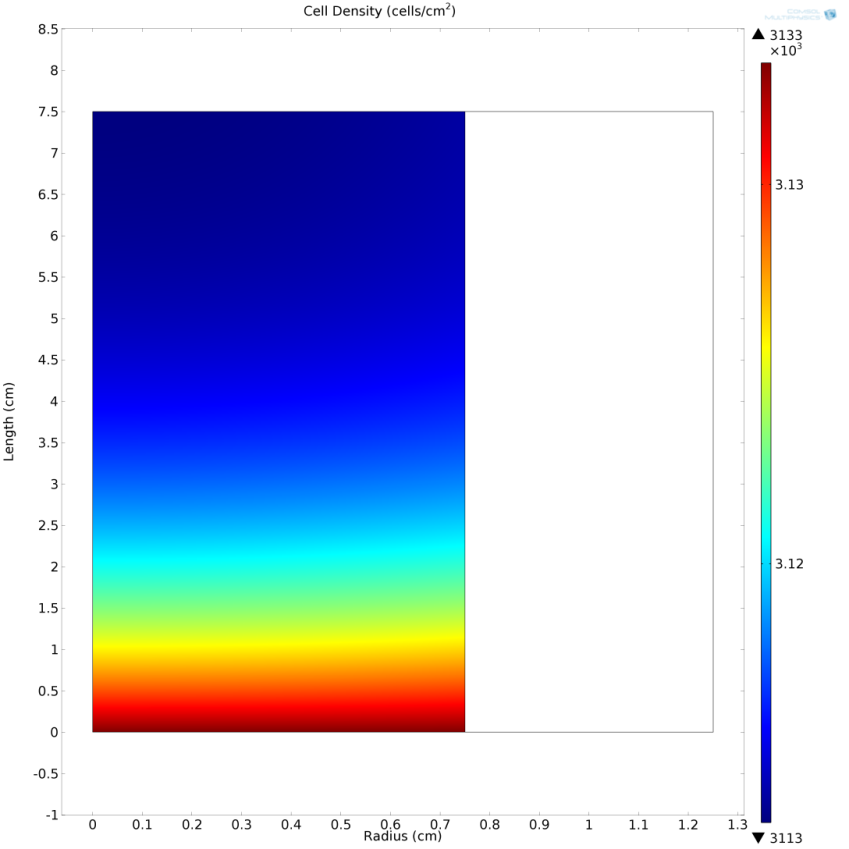


Cell density day (x10^3^ cells/cm^2^) day 4


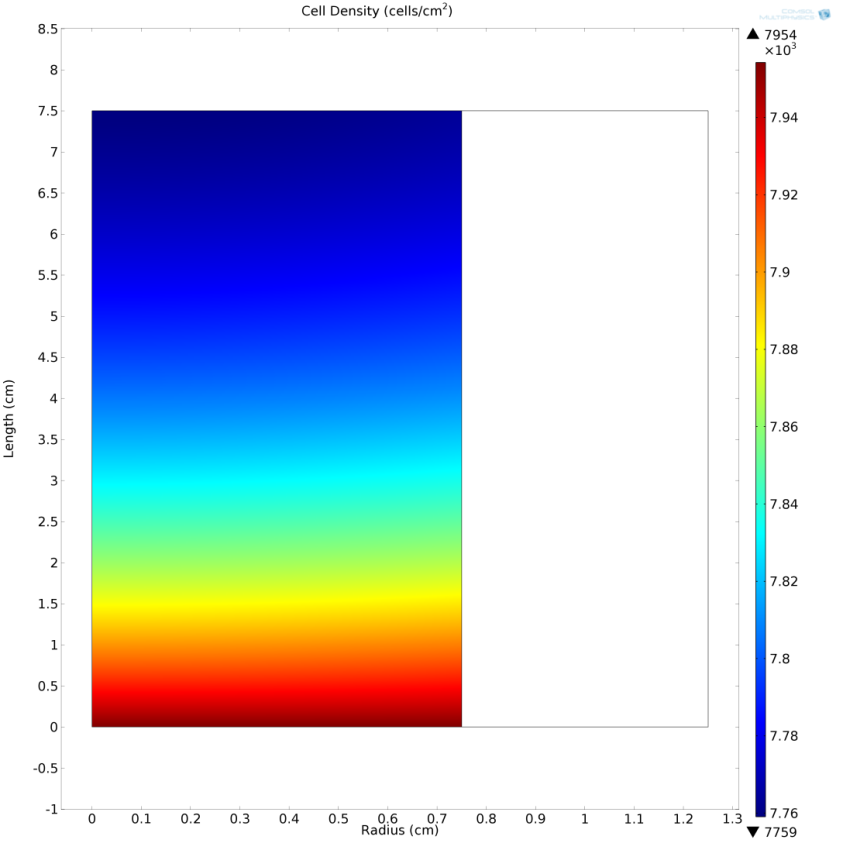


Cell density day (x10^3^ cells/cm^2^) day 6


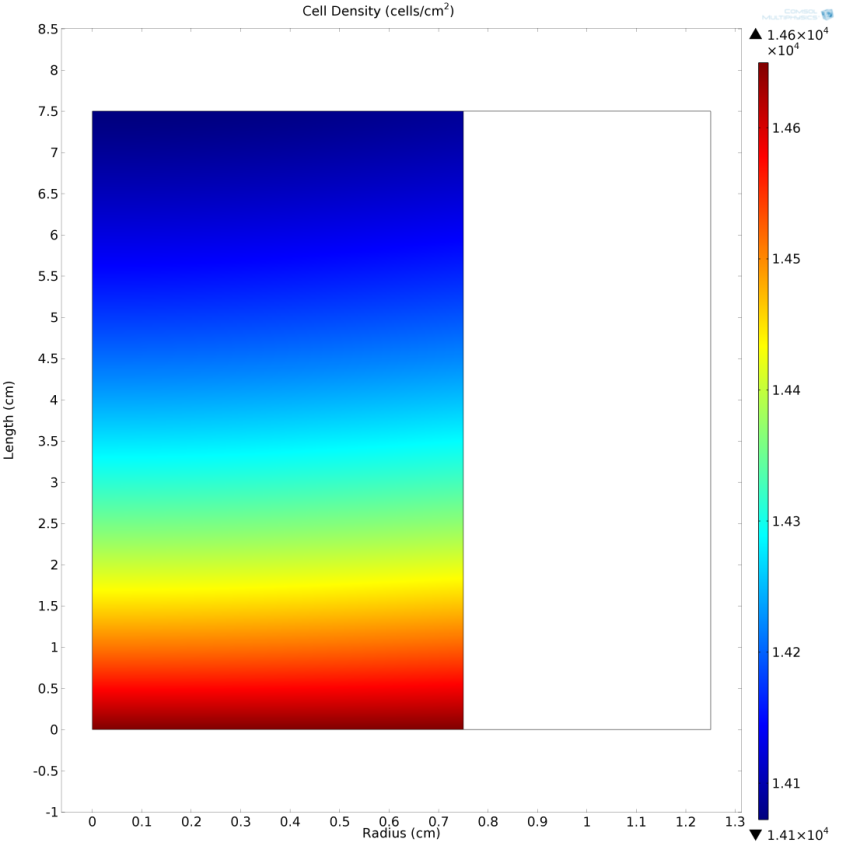


Cell density (x10^4^ cells/cm^2^) day 8


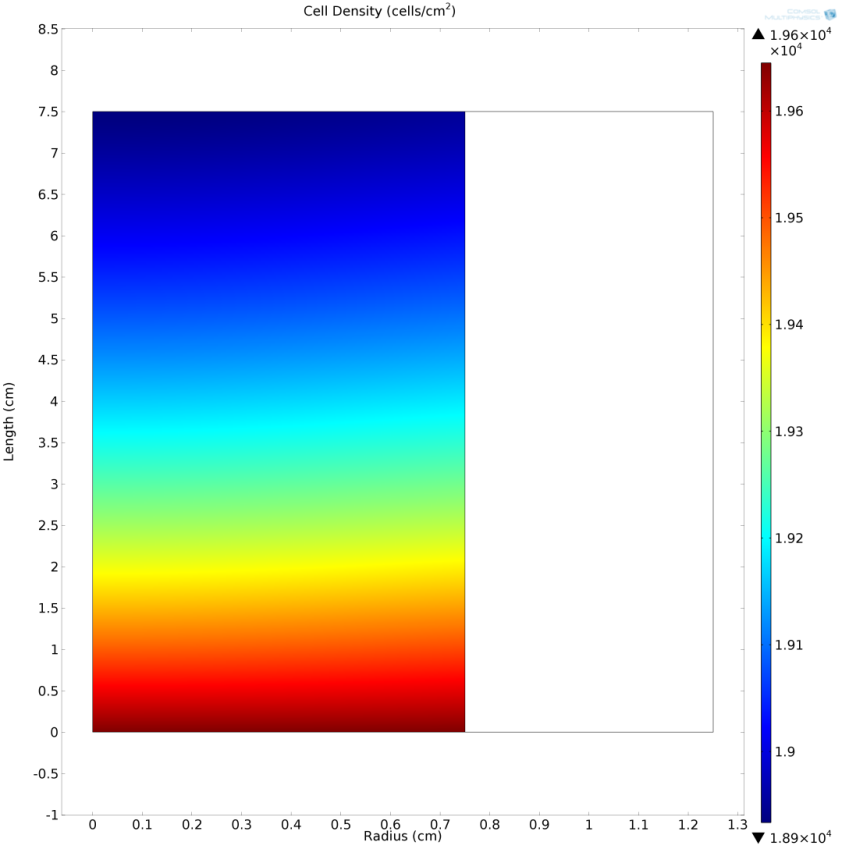

Supplement: S2 File — (DOCX) [file pone.0202079.s002.docx]
