# Supplementary material for: Modelling mesenchymal stromal cell growth in a packed bed bioreactor with a gas permeable wall
Source: PLoS One. 2018 Aug 27;13(8):e0202079. doi: 10.1371/journal.pone.0202079 (PMC6110476; doi:10.1371/journal.pone.0202079)

Predicted oxygen concentration (mM) in reactor vessel and PDMS wall of the base model. White arrows represent the direction of combined diffusive and convective flux and the black arrow represents the direction of the diffusive flux.

Oxygen concentration (mM) day 2


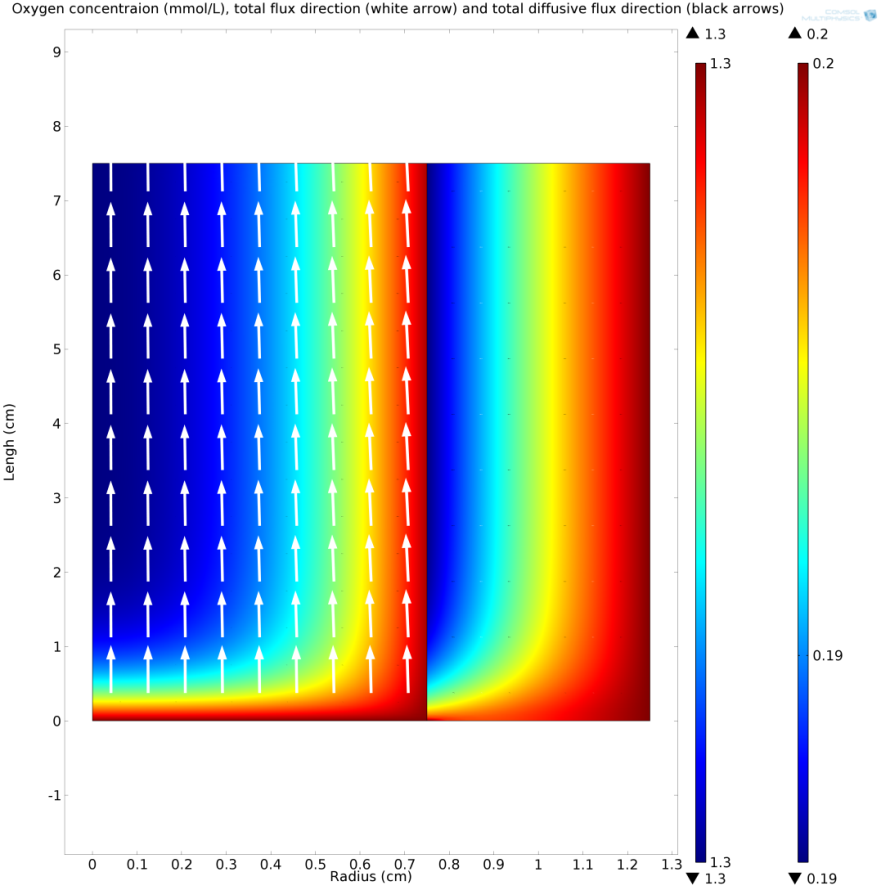


Oxygen concentration (mM) day 4


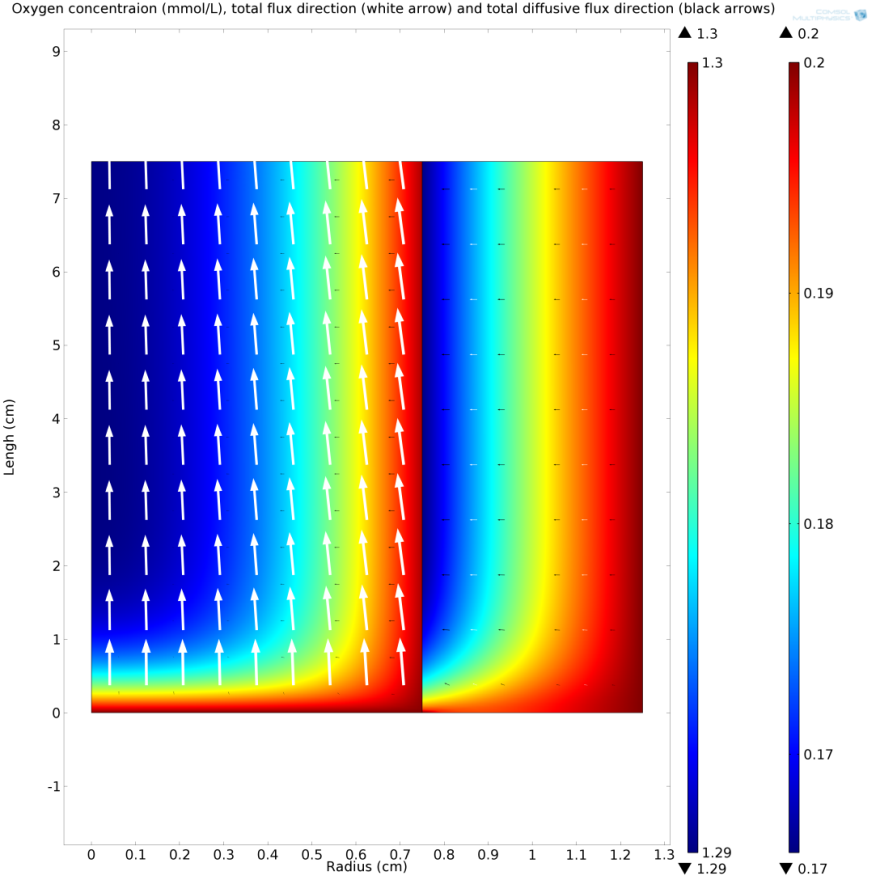


Oxygen concentration (mM) day 6


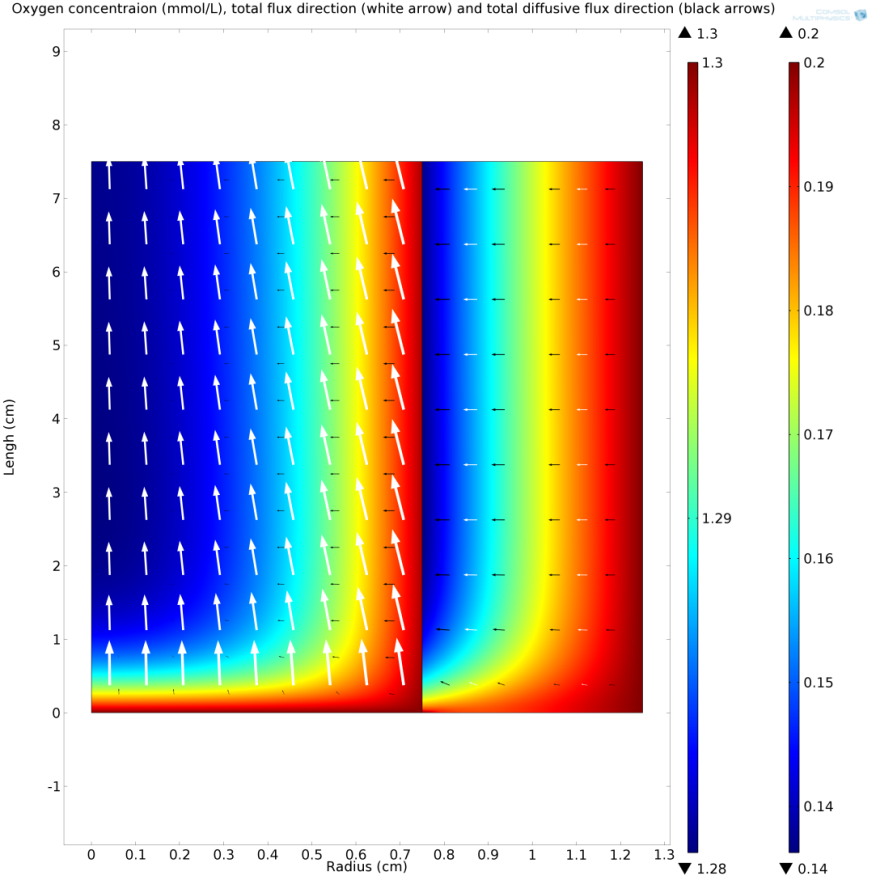


Oxygen concentration (mM) day 8


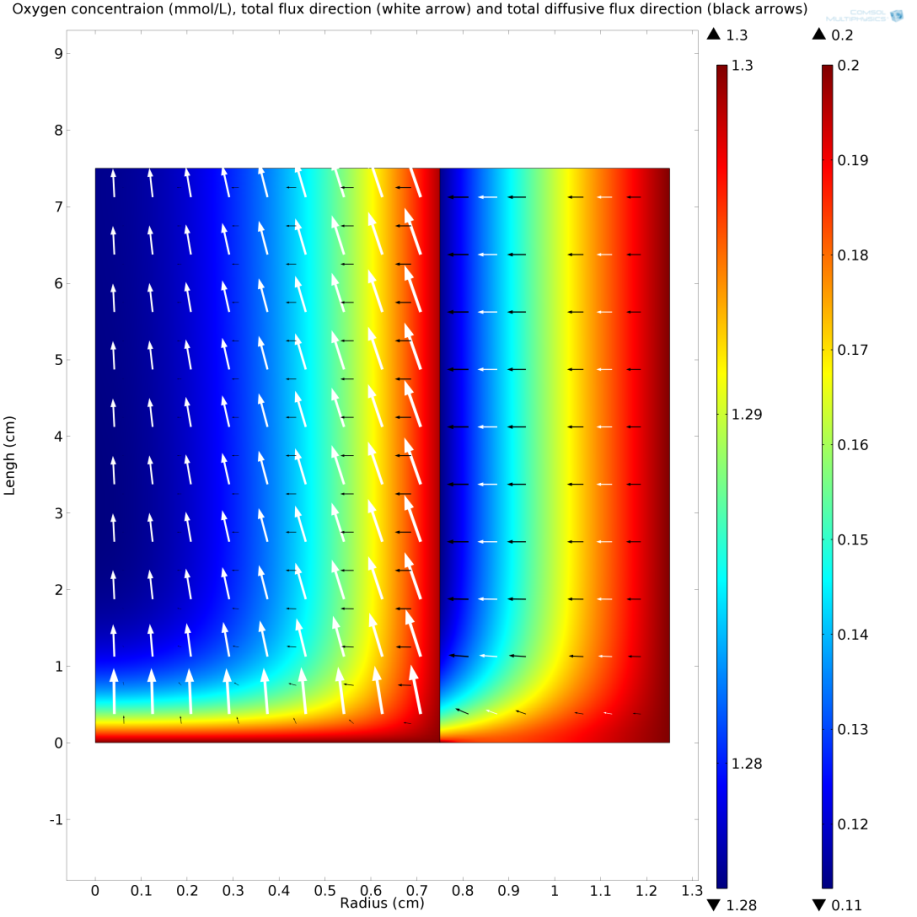

Supplement: S3 File — (DOCX) [file pone.0202079.s003.docx]
