# Supplementary material for: Modelling mesenchymal stromal cell growth in a packed bed bioreactor with a gas permeable wall
Source: PLoS One. 2018 Aug 27;13(8):e0202079. doi: 10.1371/journal.pone.0202079 (PMC6110476; doi:10.1371/journal.pone.0202079)

Glucose concentration (mM) day 2


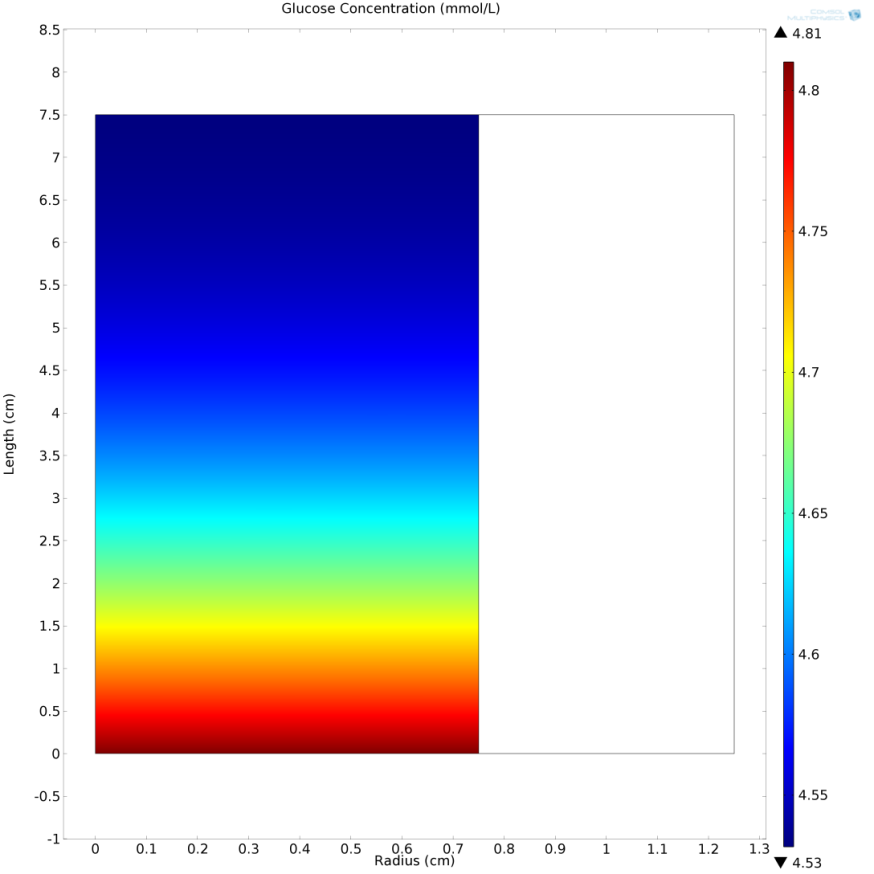


Glucose concentration (mM) day 4


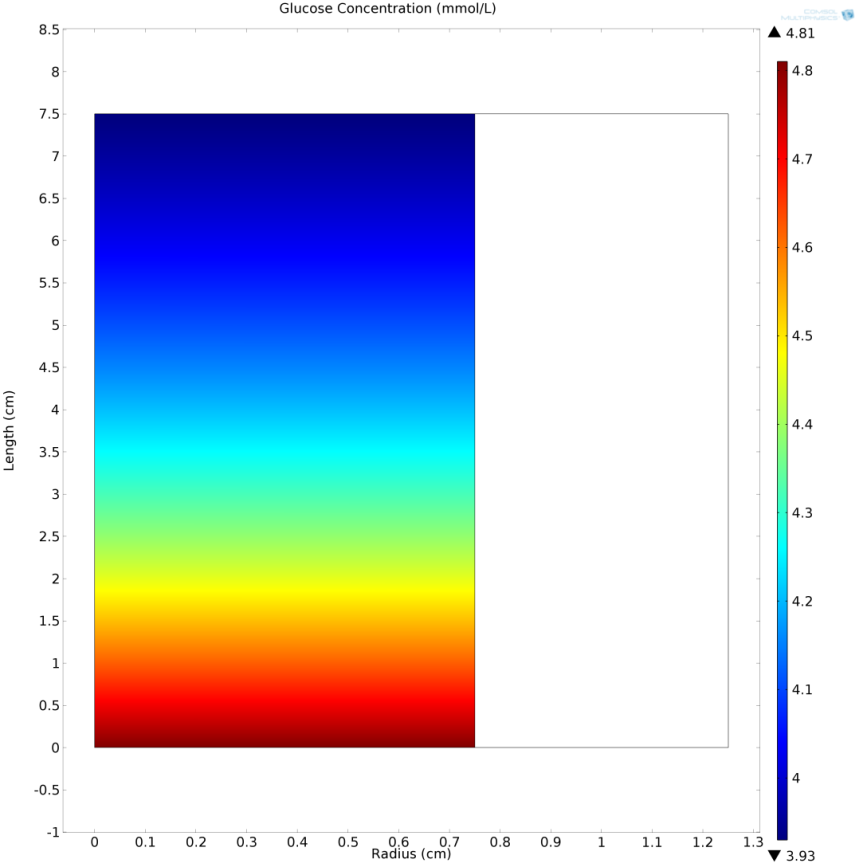


Glucose concentration (mM) day 6


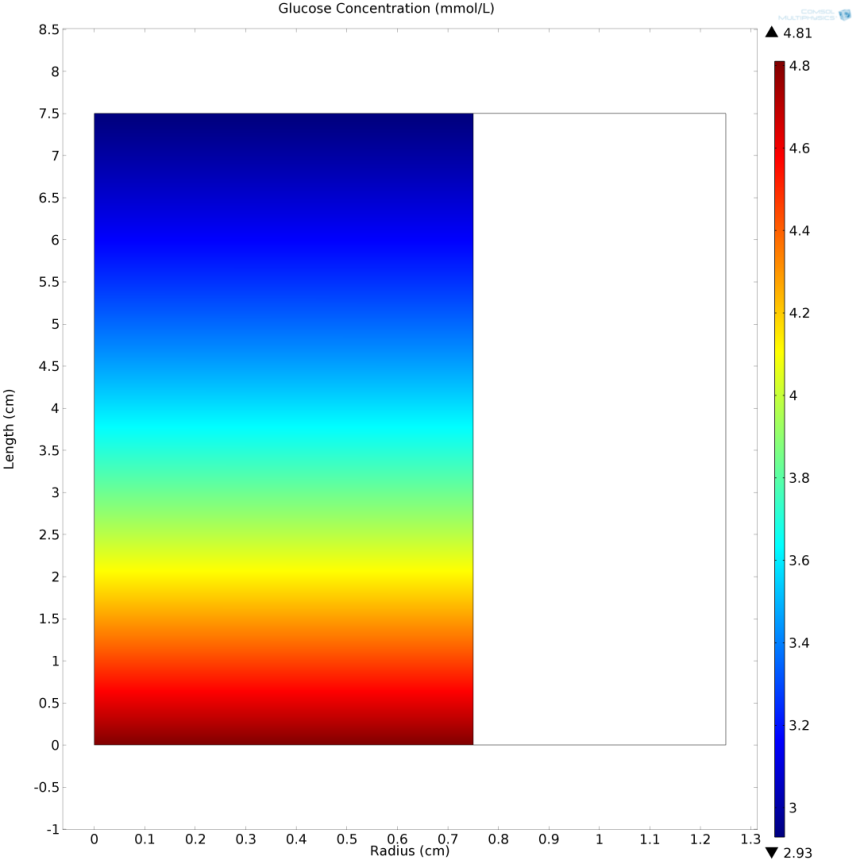


Oxygen concentration (mM) day 8


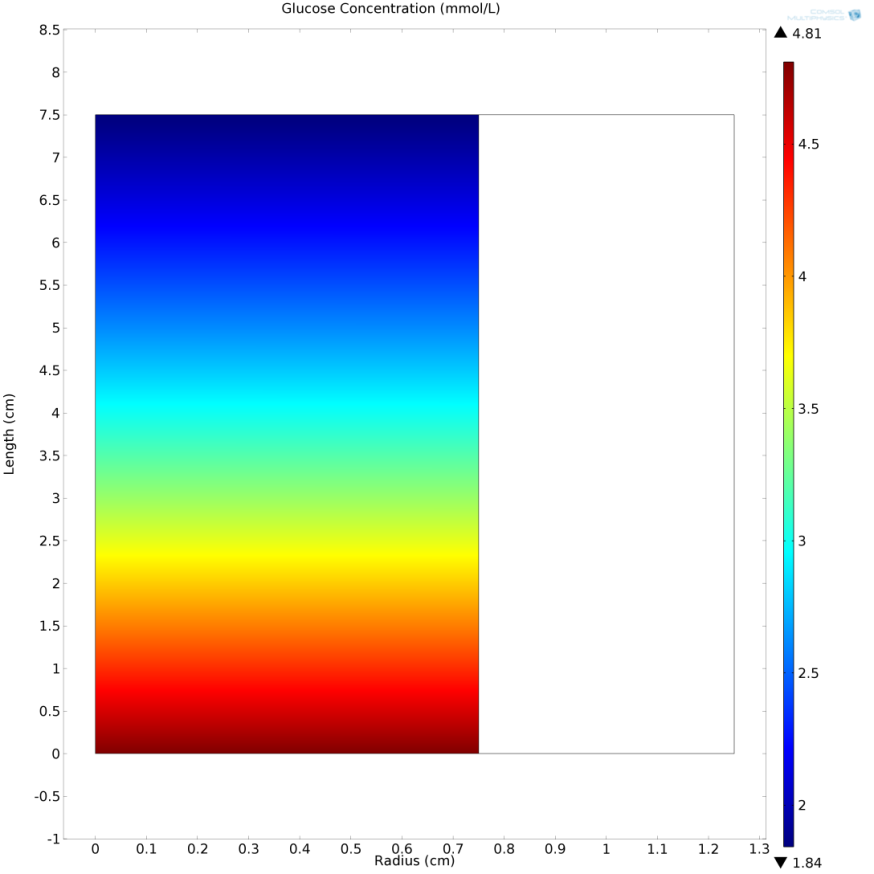

Supplement: S4 File — (DOCX) [file pone.0202079.s004.docx]
