# Supplementary material for: Modelling mesenchymal stromal cell growth in a packed bed bioreactor with a gas permeable wall
Source: PLoS One. 2018 Aug 27;13(8):e0202079. doi: 10.1371/journal.pone.0202079 (PMC6110476; doi:10.1371/journal.pone.0202079)

Lactate concentration (mM) day 2


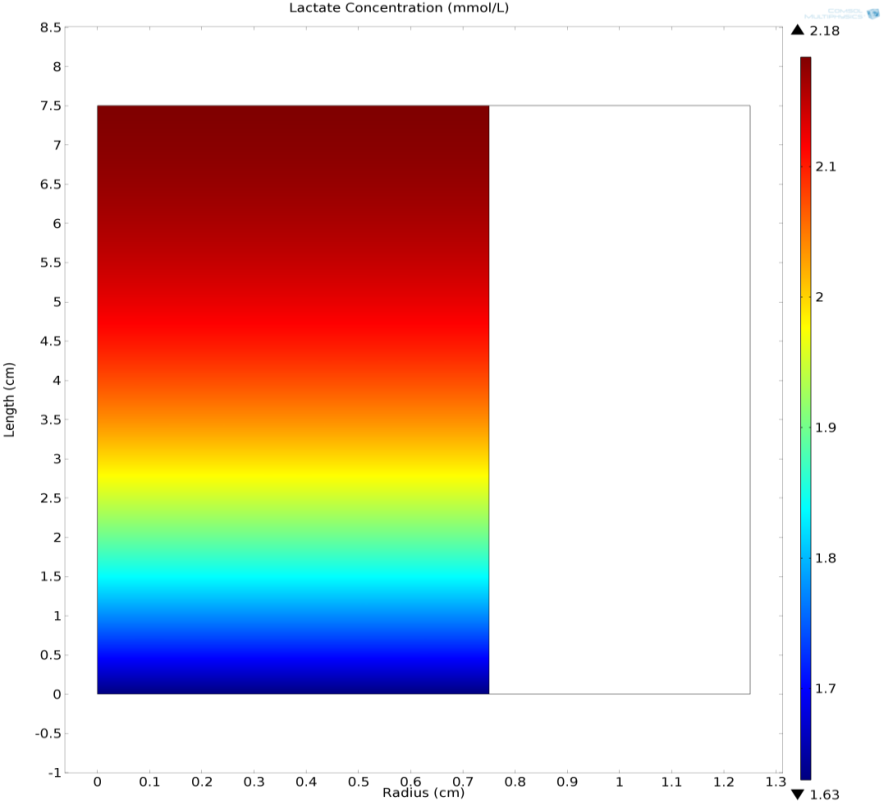


Lactate concentration (mM) day 4


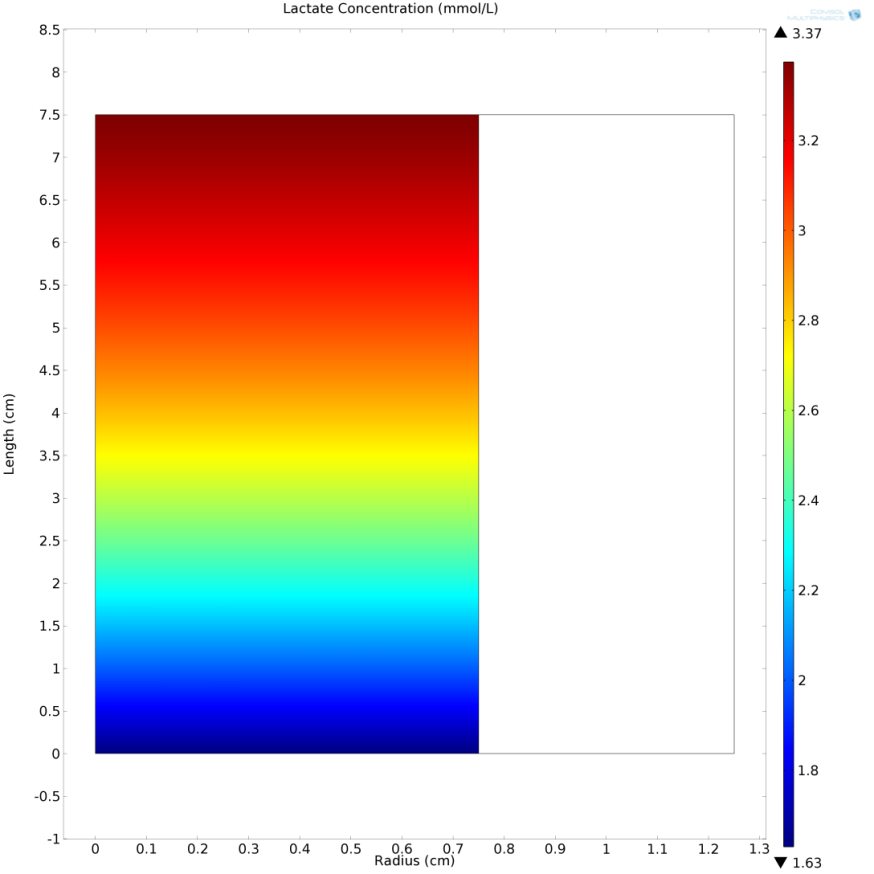


Lactate concentration (mM) day 6


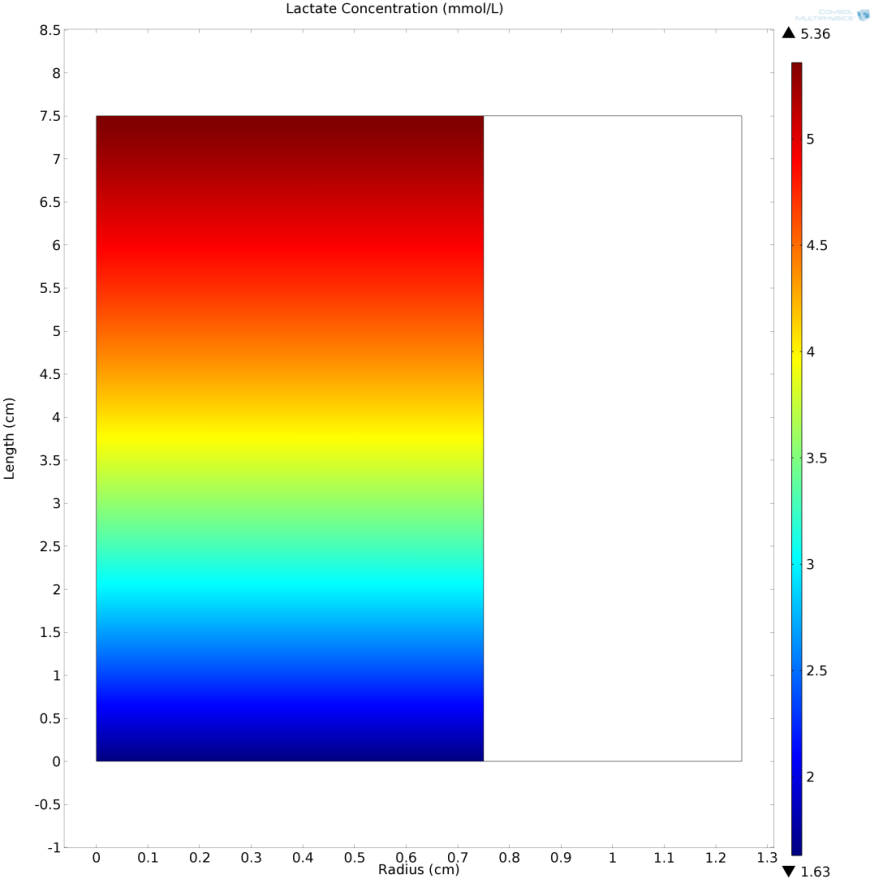


Lactate concentration (mM) day 8


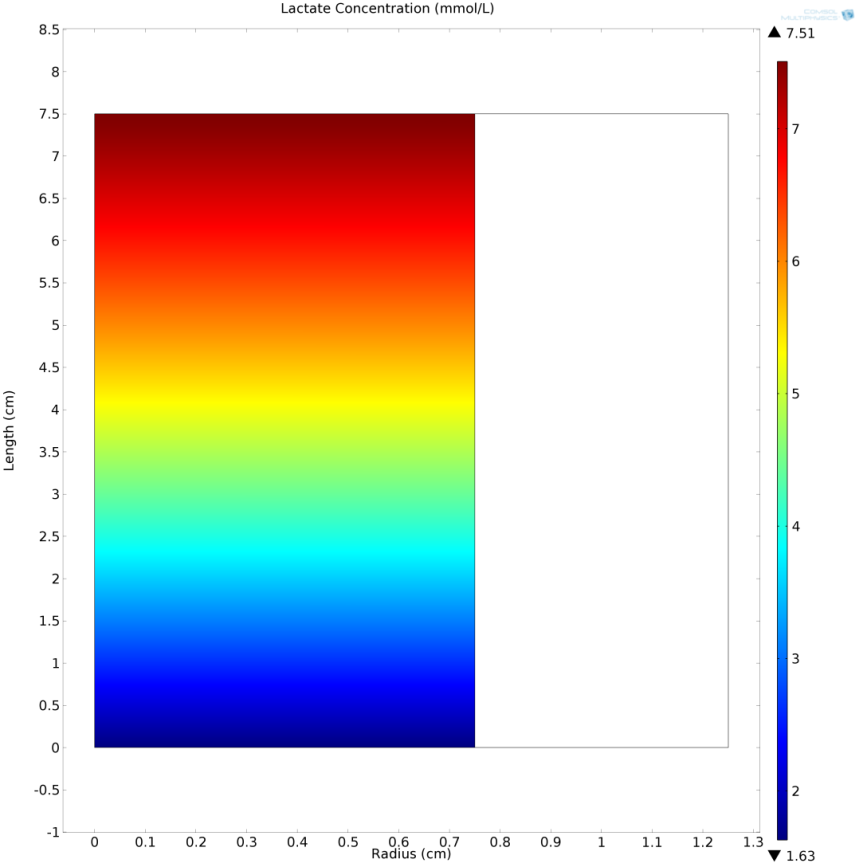

Supplement: S5 File — (DOCX) [file pone.0202079.s005.docx]
