# Supplementary material for: Modelling mesenchymal stromal cell growth in a packed bed bioreactor with a gas permeable wall
Source: PLoS One. 2018 Aug 27;13(8):e0202079. doi: 10.1371/journal.pone.0202079 (PMC6110476; doi:10.1371/journal.pone.0202079)

# Predicted oxygen concentration on the centre line of bioreactor at day 7 as the wall thickness changes

The wall does provide resistance to the oxygen flux and thus reduce the amount of oxygen available for cell growth within the bioreactor.


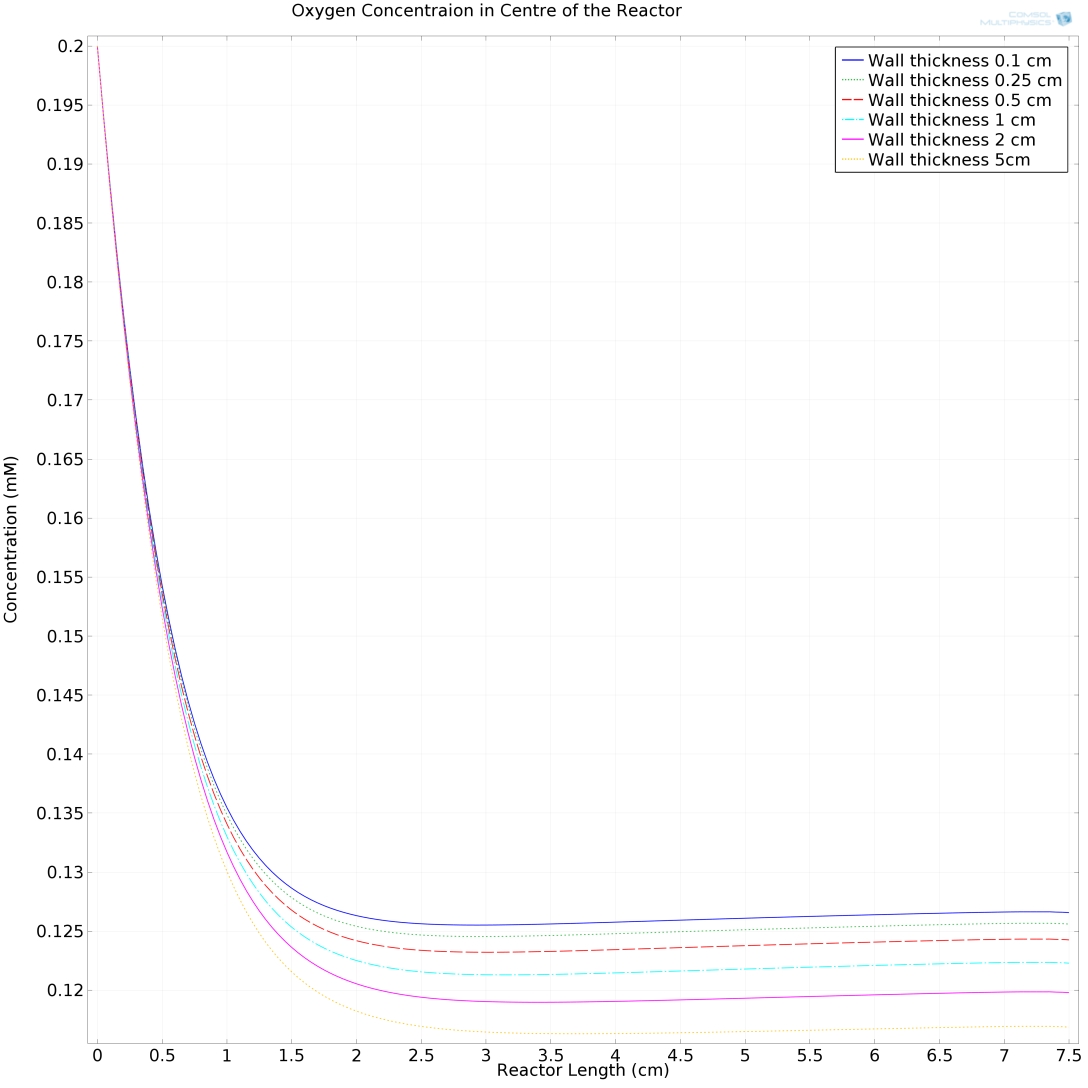


# Predicted oxygen concentration at the scaffold-wall interface


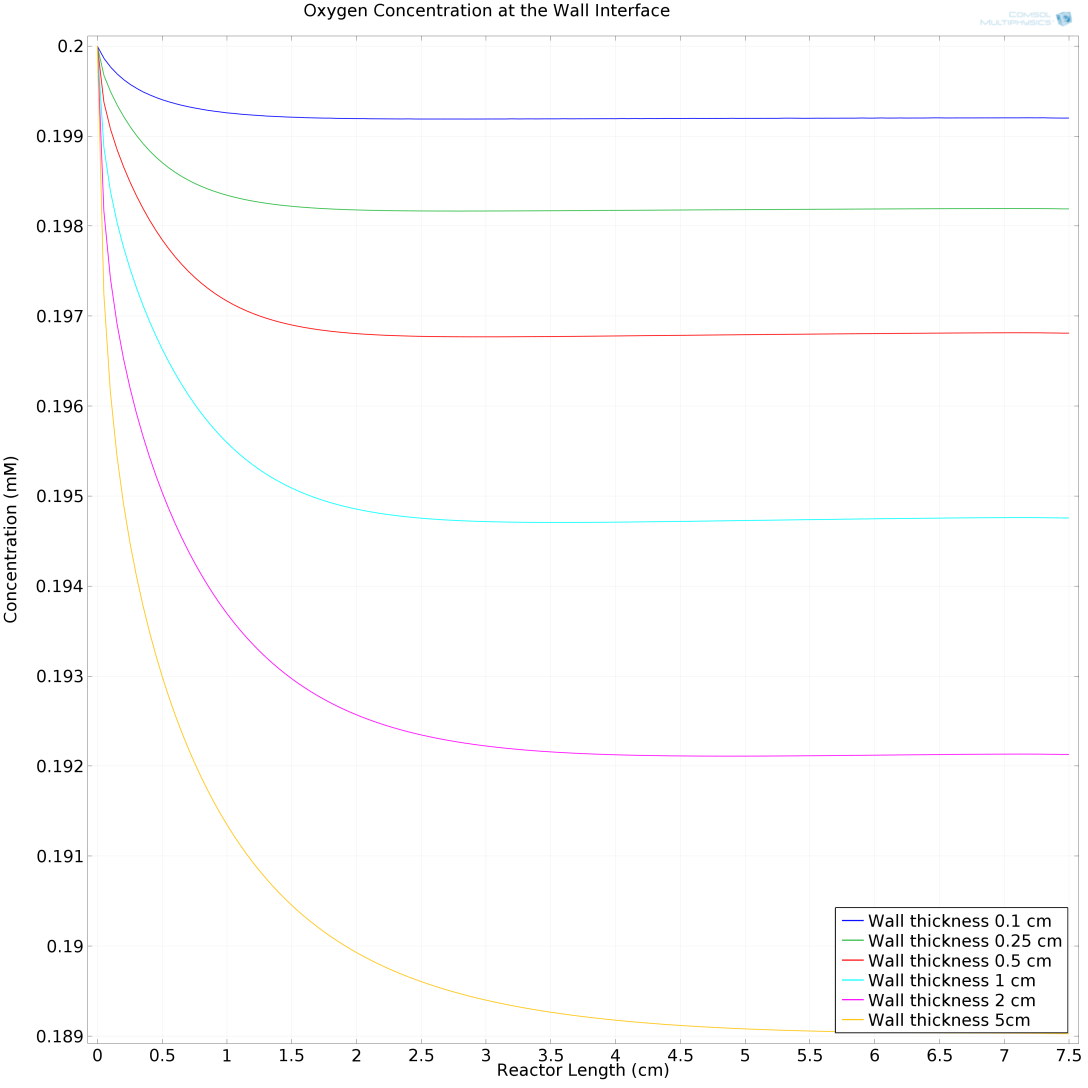

Supplement: S6 File — (DOCX) [file pone.0202079.s006.docx]
