# Supplementary material for: Modelling mesenchymal stromal cell growth in a packed bed bioreactor with a gas permeable wall
Source: PLoS One. 2018 Aug 27;13(8):e0202079. doi: 10.1371/journal.pone.0202079 (PMC6110476; doi:10.1371/journal.pone.0202079)
Supplement: S7 File — (DOCX) [file pone.0202079.s007.docx]

In order to prevent the possible upregulation of the osteogenic pathway shear experienced by the cells should not exceed 0.015 Pa.
