# Supplementary material for: Modelling mesenchymal stromal cell growth in a packed bed bioreactor with a gas permeable wall
Source: PLoS One. 2018 Aug 27;13(8):e0202079. doi: 10.1371/journal.pone.0202079 (PMC6110476; doi:10.1371/journal.pone.0202079)
Supplement: S8 File — (DOCX) [file pone.0202079.s008.docx]

A tube rocker-roller method was used to seed the MSCs in the bioreactor. 1000 green florescence-protein labelled mouse mesenchymal stromal cells/cm^2^ were seeded into the bioreactor suspended in DMEM-containing serum. To distribute the cells evenly, the bioreactor was placed on a tube rocker-roller set at 5 RPM for 10 minutes, followed by 5 minutes of rest in an incubator (37°C and 5% CO_2_) this process was repeated for 3 hr. The static method was used as a control. Here the cells were injected to the bioreactor and left to attach for 3 hr. After this, the medium was drained out and the non-adherent cells counted on an FC 500 flow cytometer (Beckman and Coulter, USA) using flow cytometry counting beads (Beckman and Coulter, USA). This number was compared to the initial cell number to calculate the seeding efficiency.
