# Supplementary material for: Modelling mesenchymal stromal cell growth in a packed bed bioreactor with a gas permeable wall
Source: PLoS One. 2018 Aug 27;13(8):e0202079. doi: 10.1371/journal.pone.0202079 (PMC6110476; doi:10.1371/journal.pone.0202079)

In our large scale experiments the bioreactor cultures where only cultured for 7 days. Our model confirms that 30 ml/hr provides adequate supply of oxygen and glucose, and the removal of lactate. However, at lower flow rates the radial diffusion of oxygen is not sufficient. With area of very low oxygen concentration in the centre of the reactor near the outlet at lower flow rates. This suggest that additional passive oxygen sources are required for larger bioreactor systems.

30 ml/hr Glucose concentration day 7


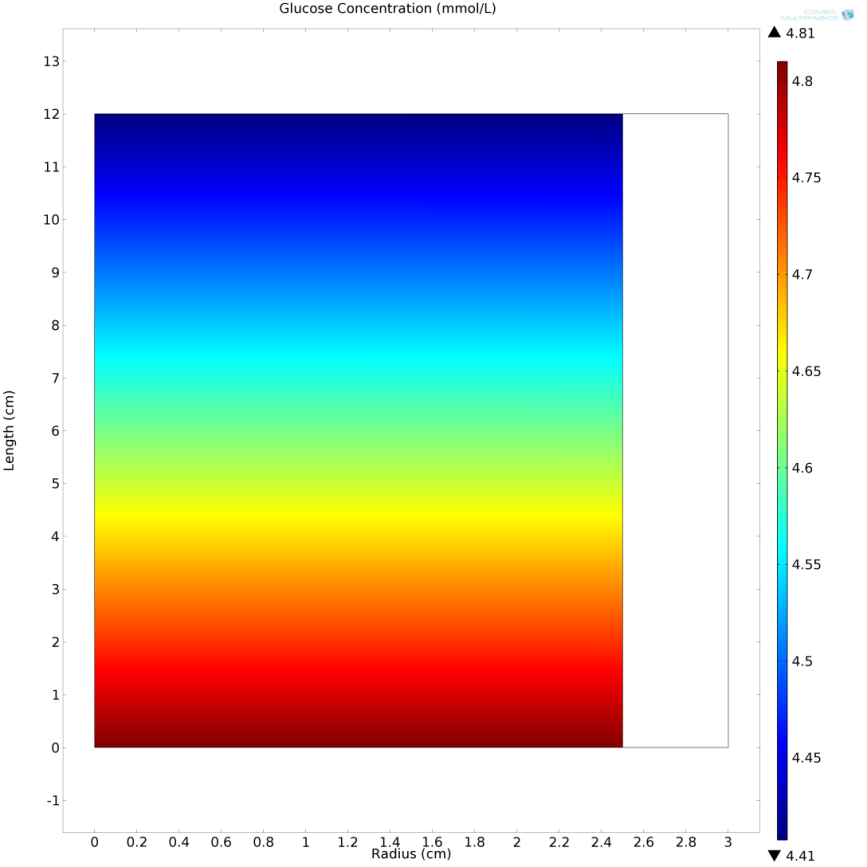


30 ml/hr Oxygen concentration day 7


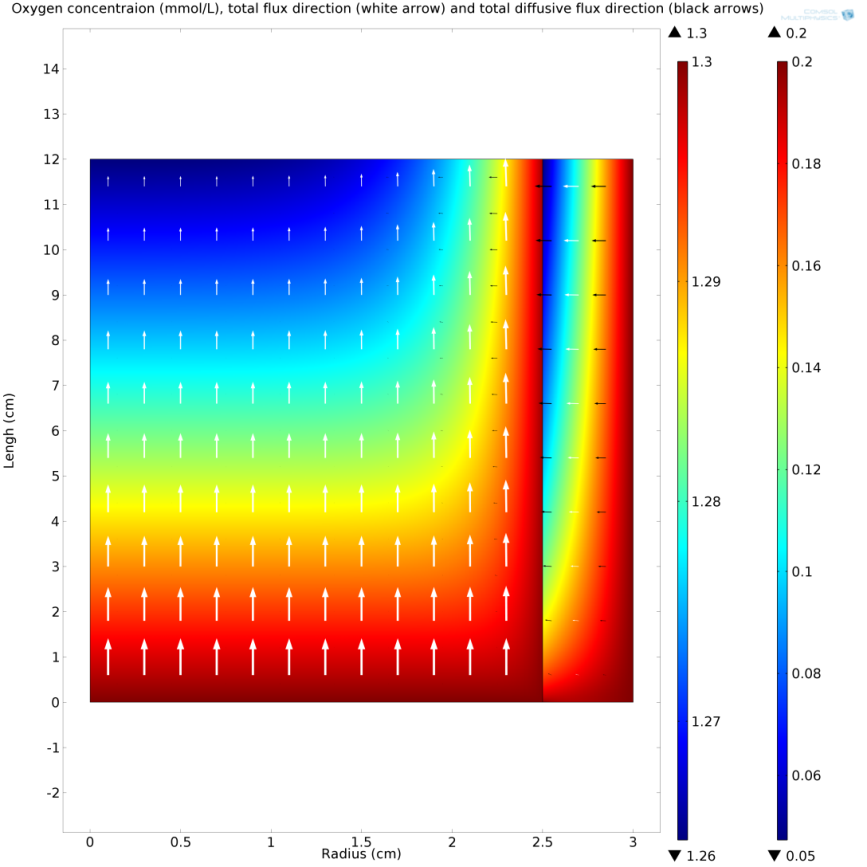


20ml/hr Glucose concentration day 7


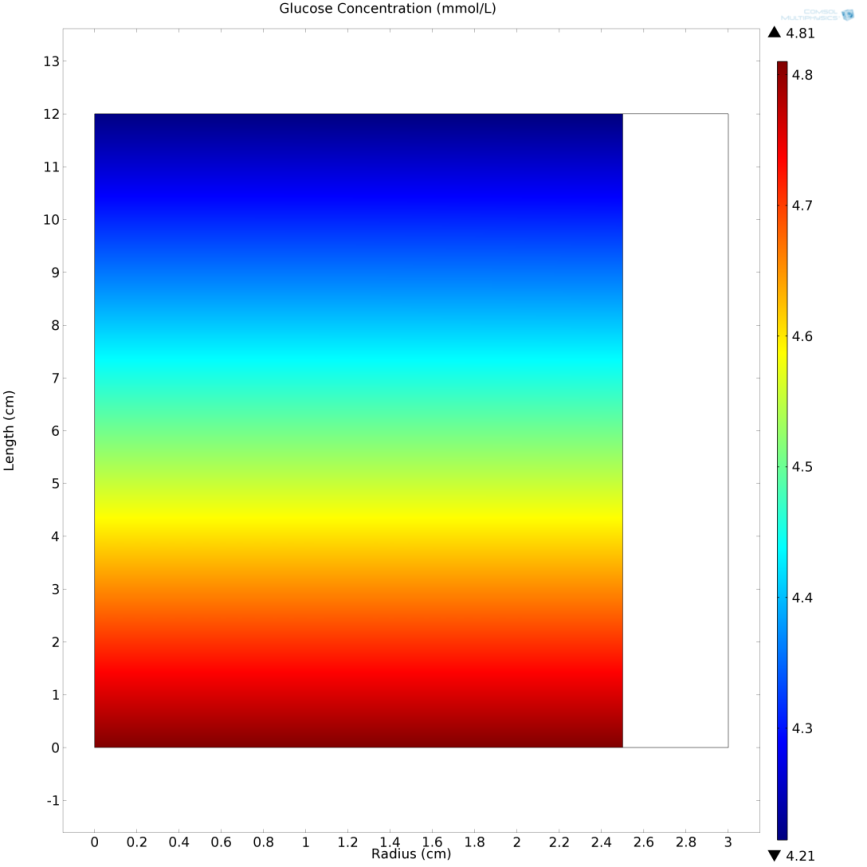


20 ml/hr Oxygen concentration day 7


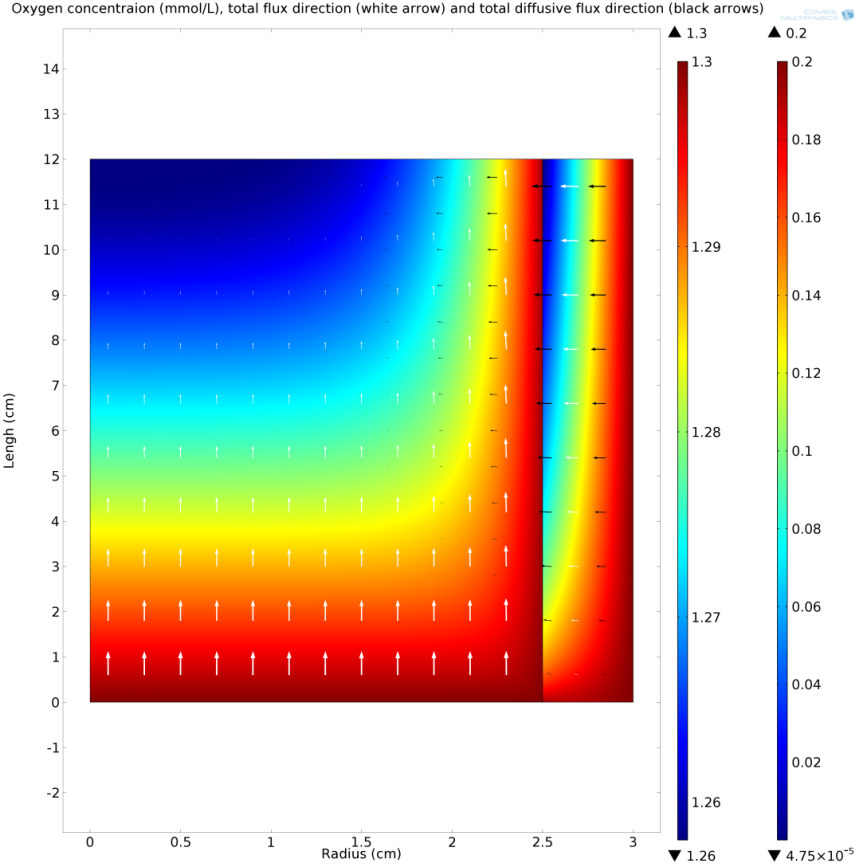


15 ml/hr Glucose concentration day 7


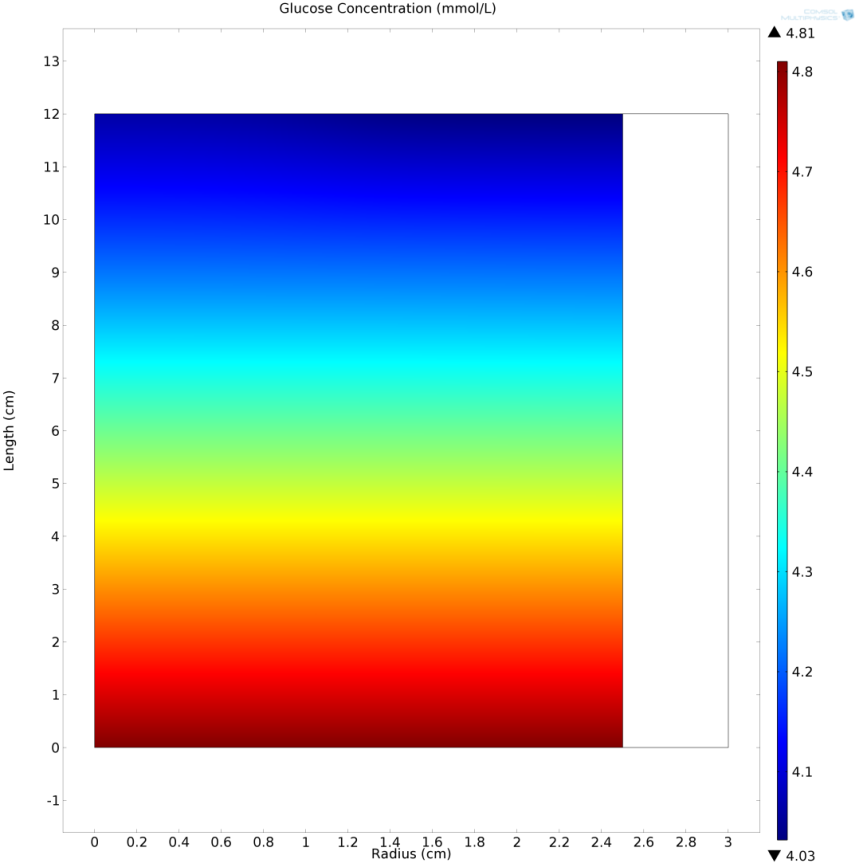


15 ml/hr Oxygen concentration day 7


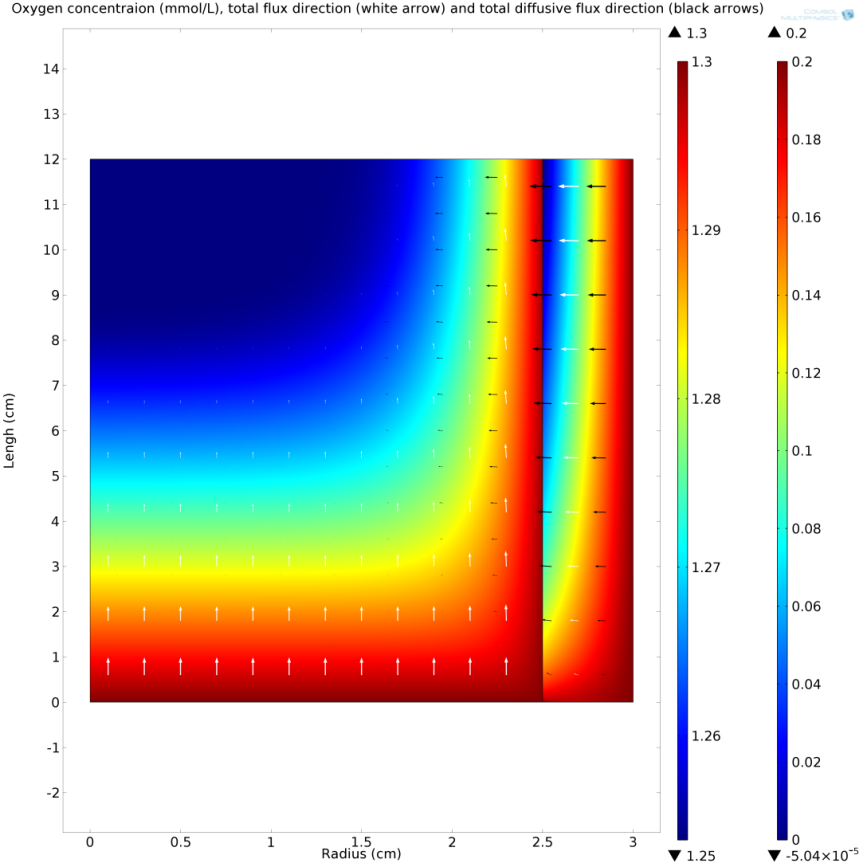


10 ml/hr Glucose concentration day 7


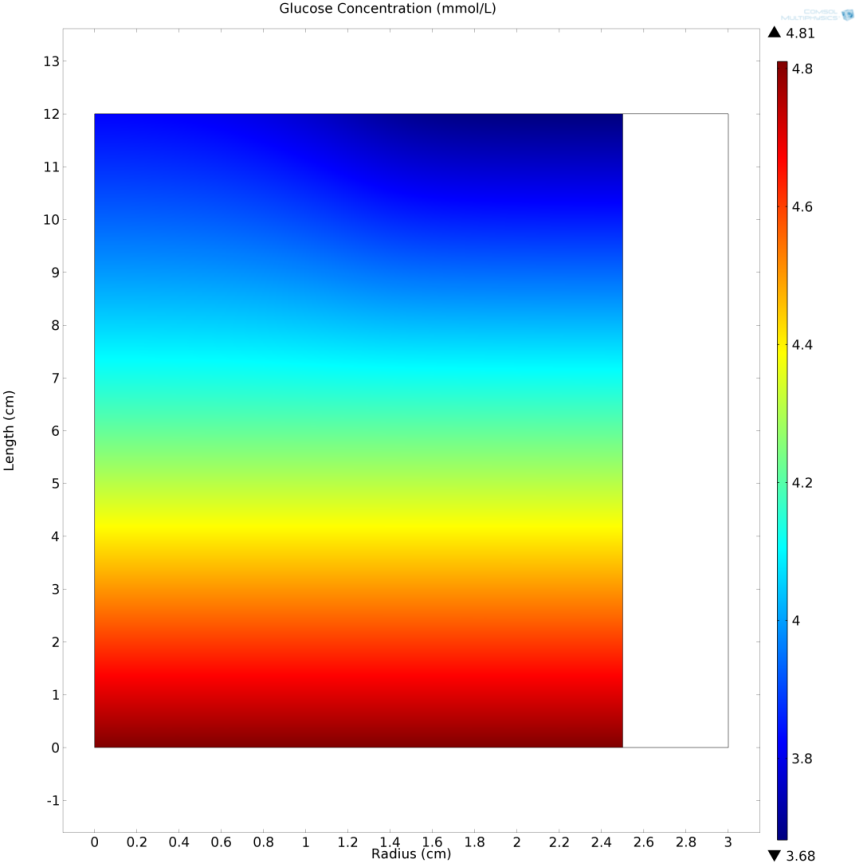


10 ml/hr Oxygen concentration day 7


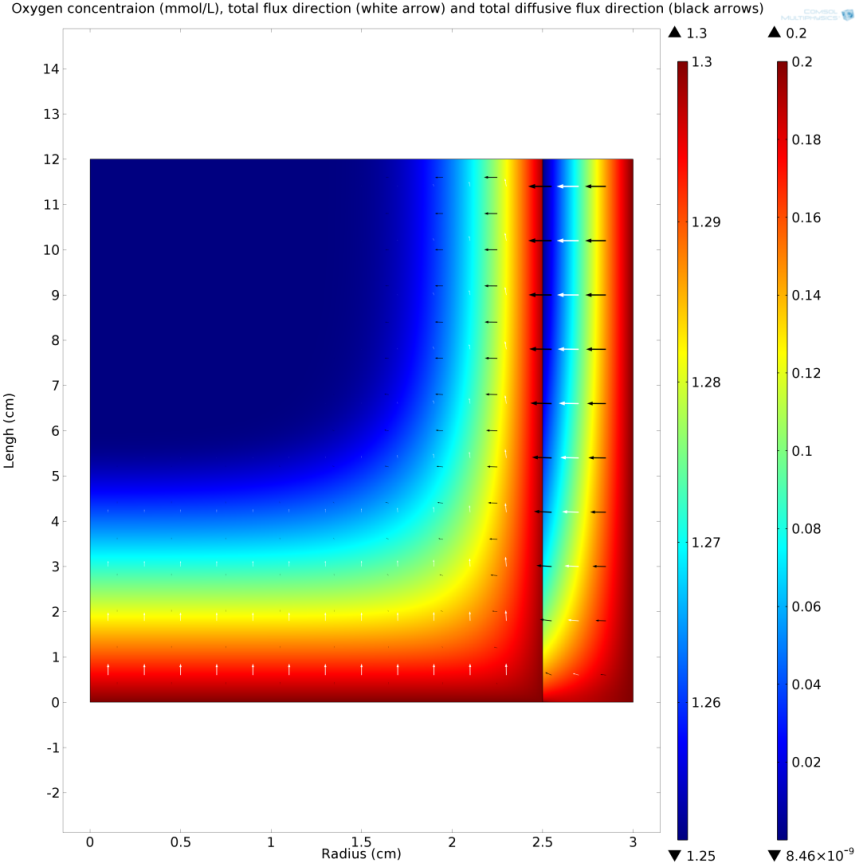

Supplement: S9 File — (DOCX) [file pone.0202079.s009.docx]
